# Analysis of high iron rice lines reveals new miRNAs that target iron transporters in roots

**DOI:** 10.1093/jxb/erw346

**Published:** 2016-10-11

**Authors:** Soumitra Paul, Dipak Gayen, Swapan K. Datta, Karabi Datta

**Affiliations:** Laboratory of Translational Research on Transgenic Crops, Department of Botany, University of Calcutta, 35 Ballygunge Circular Road, Kolkata-700019, West Bengal, India

**Keywords:** Iron transport, milk stage, miRNA, OsNRAMP4, root, soy*FER*1-overexpressing rice.

## Abstract

The present study highlights the molecular regulation of iron transport in soy*FER*1-overexpressing transgenic rice. Accumulation of iron in three different seed developmental stages, milk, dough, and mature, has been examined. The transgenic seeds of the milk stage showed significant augmentation of iron and zinc levels compared with wild-type seeds, and similar results were observed throughout the dough and mature stages. To investigate the regulation of iron transport, the role of miRNAs was studied in roots of transgenic rice. Sequencing of small RNA libraries revealed 153 known and 41 novel miRNAs in roots. Among them, 59 known and 14 novel miRNAs were found to be significantly expressed. miR166, miR399, and miR408 were identified as playing a vital role in iron uptake in roots of transgenic plants . Most importantly, four putative novel miRNAs, namely miR11, miR26, miR30, and miR31, were found to be down-regulated in roots of transgenic plants. For all these four novel miRNAs, *natural resistance-associated macrophage protein* 4 (*NRAMP*4), encoding a metal transporter, was predicted as a target gene. It is hypothesized that the NRAMP4 transporter is activated in roots of transgenic plants due to the lower abundance of its corresponding putative novel miRNAs. The relative transcript level of the *NRAMP*4 transcript was increased from 0.107 in the wild type to 65.24 and 55.39 in transgenic plants, which demonstrates the elevated amount of iron transport in transgenic plants. In addition, up-regulation of *OsYSL*15, *OsFRO*2, and *OsIRT*1 in roots also facilitates iron loading in transgenic seeds.

## Highlight

Novel miRNAs regulating NRAMP4 activity in root induce iron accumulation in ferritin-overexpressing rice grains during the milk stage of seed development. Enhancement of YSL15, IRT2, and FRO2 facilitates this process.

## Introduction

Iron is an essential mineral nutrient required for human physiology. A low intake of iron through food or low iron bioavailability causes nutrient disorders such as iron deficiency anaemia (IDA) which is commonly observed in the Indian population consuming rice as a major food (Stein *et al.*, 2008). Recently, several biotechnological approaches have been exploited to increase the iron content in milled rice grain (Kobayashi *et al.*, 2014). Endosperm-specific overexpression of various transporters and enzymes involved in phytosiderophore biosynthesis such as ferric chelate reductase oxidase 2 (FRO2), iron-related transporter 1 (IRT1), yellow stripe-like (YSL) family members, NAS, and NAAT has increased iron in milled rice grain by 3- to 4-fold (Wang *et al.*, 2013). Tissue-specific overexpression of rice endogenous ferritin (*OsFER*2) or soybean ferritin (soy*FER*1) has been shown to increase iron in the endosperm (milled rice) by 2.54- and 2.09-fold, respectively (Vasconcelos *et al.*, 2003; Paul *et al.*, 2012). Ferritin stores ~4500 ferric ions in its cavity. Overexpression of ferritin in rice endosperm activates several iron transporters which facilitates iron and zinc loading in rice endosperm (Paul *et al.*, 2012).

In monocots such as rice, ferric ions are chelated in plant roots by mugineic acid (MA) family members such as nicotianamine, deoxymugineic acid (DMA), and 3-hydroxymugineic acid (3-HMA) and are transported to seeds via YSL transporters (Kim and Guerinot, 2007). In contrast, ferrous ions are transported by pathways mediated by different natural resistance-associated macrophage proteins (NRAMPs) and members of the IRT family (Kim and Guerinot, 2007). Deficiency of iron in soil triggers the expression of different groups of transporters in roots to maintain plant iron homeostasis (Kobayashi *et al.*, 2014). The activated transporters or importers stimulate uptake of iron or other divalent cations such as zinc from the soil and mobilize them into different compartments. During different seed developmental stages in transgenic *NAS*-, *ferritin*-, and *phytase*-overexpressing plants (NFPs), variations in the expression of 28 genes involved in iron homeostasis was observed depending on the soil iron content (Wang *et al.*, 2013). These transgenes were able to induce the expression of YSL, IRT, and members of other families of transporters in the roots, flag leaves, and seeds of the transgenic plants to facilitate iron uptake and mobilization. A recent study has shown that during iron-deficient conditions, 24-epibrassinolide (EBR) increases the expression of several iron transporters in roots and suppresses them in shoots, thereby regulating iron homeostasis (Wang *et al.*, 2015). However, the underlying molecular mechanisms regulating iron homeostasis in plants remain obscure.

MiRNAs, members of the small RNA family, are vital in regulating diverse physiological processes such as growth and development, biotic and abiotic stress responses, and homeostasis of various nutrients in plants (Sunkar *et al.*, 2012; Paul *et al.*, 2015; Zhang, 2015). In Arabidopsis, the miRNA families miR159, miR167, miR172, miR173, and miR394 are iron deficiency responsive (Waters *et al.*, 2012). However, the role of miRNAs in regulating nutrient transporters in high iron transgenic plants has not been explored. MiRNAs with their near perfect complementarities bind to target gene transcripts and create dsRNAs that are subsequently cleaved, thereby silencing expression of the gene (Mallory and Bouche, 2008). Depending on the nutrient condition (high or low), the nutrient stress-responsive miRNAs control nutrient transport by modulating the expression of nutrient transporters via suppression of signalling molecules/transcription factors involved in regulating expression of transporters (Paul *et al.*, 2015).

Since miRNAs are genotype dependent and transgene integration may alter the miRNA expression profile, we used two independent homozygous soy*FER*1-overexpressing transgenic rice lines (TF1 and TF2) to validate the expression of the miRNAs. The miRNAs from TF1 and TF2 roots were analysed during different seed developmental stages to understand their role in the iron uptake mechanism in iron-enriched plants. We prepared two root-specific small RNA libraries from wild-type (WT) and TF1 plants and sequenced them.

A total of 153 conserved and 41 novel miRNAs were identified from those two libraries. The roots of the WT and TF1 showed 28 and 20 novel miRNAs, respectively, among which seven were found to be common to both. Fourteen novel miRNAs were considered as significant. The attenuated level of four putative novel miRNAs, namely miR11, miR26, miR30, and miR31, in the roots of transgenic plants was significantly associated with the elevation of one key iron transporter, NRAMP4.

## Materials and methods

### Plant materials and growth

Rice (*Oryza sativa* L. subspecies *indica*) cv. IR68144 and soy*FER*1-overexpressing homozygous independent transgenic lines of IR68144 (Vasconcelos *et al.*, 2003) were selected as WT and TF lines, respectively. In the transgenic plants, the soy*FER*1 gene (780bp) was overexpressed under the control of the endosperm-specific *OsGLUB*1 promoter (1.2kb). The plant harbours the *bar* gene as the selective marker. The homozygous plants from two independent transgenic lines, TF1 and TF2, were selected. The plants were grown in fertilizer-enriched (N:P:K=80:40:40kg ha^–1^) paddy field soil under greenhouse conditions with a day/night temperature regime of 30/ 25 °C under natural illumination and a relative humidity of 70–80%. The roots, flag leaves, and developing seeds (brown rice at maturity) were harvested from both lines during three seed developmental phases, namely milk, dough, and mature stages. The parameters of the different stages were selected according to the Rice knowledge Bank, IRRI, Philippines. The samples were collected with three biological replicates and used for metal concentration analysis. All the samples from different stages were stored in RNAlater solution for small RNA library sequencing purposes and kept at −80 °C.

### Analysis of soil nutrient contents

The micronutrient concentrations of soil were analysed by SGS India Pvt. Ltd. The soil of the paddy field was autoclaved and dried in a forced air drying cabinet at 35 °C. The diethylene triamine pentaacetic acid (DTPA) method of soil analysis was followed (Lindsay and Norvell, 1978). The soil was mixed twice with 5mM DTPA, pH 7.3, and shaken for 2h. The extracted micronutrients were measured using atomic absorption spectroscopy. The organic content, pH, and macronutrient concentrations (nitrogen, phosphorus, and potassium) of the soil were also analysed. Three technical replicates have been considered for soil analysis.

### Iron and zinc concentration analysis by atomic absorption spectroscopy

The roots, flag leaves, and seeds collected from WT, TF1, and TF2 plants during the three different seed developmental stages were weighed (2g) and digested using a modified protocol of dry-ashing digestion (Jiang *et al.*, 2007). The acidic ash solution was filtered through Whatman no. 42 filters, and the final volume was brought up to 25ml. The iron and zinc content of the clear filtrate was analysed using an atomic absorption spectrometer (AAnalyst200, Perkin Elmer, USA) with hollow cathode lamps (HCLs, PerkinElmer) at their respective wavelengths of 248.3nm and 213.9nm. Three plants from each line were selected for micronutrient concentration analysis. The concentration of iron and zinc was also measured three times.

### Small RNA library preparation and sequencing

The roots from TF1 plants were collected during the milk stage that exhibits the highest iron uptake efficiency and were used for the preparation of small RNA libraries. Total RNA was isolated from roots of WT and TF1 plants using TRiZol reagent (Invitrogen, USA). Three independent biological replicates of roots from WT and TF plants were used for RNA preparation. The quality and quantity of total RNA, isolated from each sample, were analysed using a Bioanalyser 2100 (Agilent Technologies). The RNA from two samples (in three replicates) was used for small RNA library preparation using a Small RNA Sample preparation Kit (Illumina Technologies) following the manufacturer’s instructions. The small RNA libraries from two types of roots (WT and TF1) were sequenced using a Hiseq Illumina 1.5. The sequence data obtained as FASTQ files were assessed for qualitative analysis using FastQC version 3 and Fastx-toolkit, version 0.0.13.

### Data mining and identification of novel miRNAs

The raw sequences generated from the two small RNA libraries were trimmed for removal of adaptor/primer contamination and poly(A) tails using an miRDeep adaptor filter. The sequence data were pre-processed and cleaned of contaminants including low quality reads. The unique reads containing 17–23 nucleotide long sequences from two samples were retained for genome mapping. The filtered reads from each sample were screened against non-coding RNA sequences, tRNA, rRNA, and chloroplast sequences found in the rice genome database (IRGSP-1.0.24). The reads homologous to these sequences were discarded. The conserved miRNA sequences were identified using miRplant, version 3 and mapped on to plant (rice) miRNAs (miRPlant, version 3.0) using the Bowtie alignment tool (Li and Durbin, 2009; An *et al.*, 2014). A maximum of two mismatches were considered for analysis. The novel miRNA sequences were identified by mapping of the remaining reads on to rice genome sequences using Bowtie and putative precursor sequences analysed from each alignment read. The secondary structures of novel miRNAs were predicted using RNAfold software, and genome mapping of miRNAs was processed using the plant-specific miRDeep-P core algorithm (Meyers *et al.*, 2008). The miRNA sequence data have been submitted to the NCBI SRA database under the Bioproject ‘Translational research on transgenic rice’ (accession no. PRJNA307629).

### Target gene prediction of novel miRNAs

To identify the regulation of differential gene expression for known and novel miRNAs, edgeR (R package, version 3.8.3) software was used. The target gene of novel miRNAs was predicted using the psRobot server with default parameters (Srivastava *et al.*, 2014). The software used for analysis of target gene sequences depends on reverse complementarities and target site accessibility of miRNAs based on the minimum free energy required to open the secondary structures. The locus and function of target genes were identified by analysing the target gene sequences with *O. sativa* var. *indica* cDNA, provided by The Institute for Genomic Research (TIGR; Rice Genome Annotation Project, 7.0). For single miRNAs, we found several putative target genes. Among them, a minimum penalty score of ≤2.5 exhibiting high specificity was considered for the identification of a specific target gene.

### Validation of miRNA expression by qRT–PCR

The putative novel and known miRNAs (from small RNA sequencing data) were selected for quantitative reverse transcription–PCR (qRT–PCR)-mediated expression validation. For miRNAs, TRizol-mediated (Invitrogen, USA) RNA isolation was carried out according to the manufacturer’s instructions. The concentration of RNA was measured using a Nanodrop spectrophotometer (Bio-Rad, USA). cDNA was prepared using an NCode VILO miRNA cDNA synthesis kit (Invitrogen, USA) according to the manufacturer’s protocol. The stem–loop reverse transcription primers and forward primers for novel miRNAs were designed from aligned and identified precursor miRNAs sequences using miRNA primer designer software (Supplementary Table S1 at *JXB* online). The forward primers of known miRNAs were obtained from rice miRNA data found in miRbase. The qRT–PCR was performed using SYBR Green (Fermentas, USA) and the cycle was as follows: 95 °C for 30s, 59 °C for 30s, and 72 °C for 30s. The procedure was according to the manufacturer’s instructions (CFX 96 Real time system, Bio-Rad). The quantitative variation between different samples was evaluated by the ΔΔCt method (Livak and Schmittgen, 2001), and the ampliﬁcation of the *U6* small RNA gene was used as the internal control to normalize all data. To validate the results, three plants from each line (WT, TF1, and TF2) were selected as three biological replicates, and roots, leaves, and seeds were collected from each plant.

### Expression analysis of target genes and transporters by qRT–PCR

The target genes of miRNAs and transporter genes were selected for qRT–PCR analysis to validate the expression. Total RNA was extracted from roots, flag leaves, and developing grains of individual plants of two lines (WT and TF) using a plant RNA mini kit (Qiagen, USA). Three biological replicates were considered from each set. The concentration of RNA was measured using a spectrophotometer (Smartspec, Bio-Rad). The cDNA was prepared using the verso cDNA synthesis kit (Thermo Scientific, USA) for gene expression analysis. The transcript sequences of miRNA target genes and transporters were found in the NCBI database. The primer sequences were designed using Primer 3 software. The qRT–PCR was performed with transcript-specific primers (Supplementary Table S2) using SYBR Green (Fermentas, USA) and the cycle were as follows: 95 °C for 30s, 59 °C for 30s, and 72 °C for 30s. The procedure was according to the manufacturer’s instructions (CFX 96 Real time system, Bio-Rad). The quantitative variation between different samples was evaluated by the ΔΔCt method, and the ampliﬁcation of the *β-tubulin* gene (LOC4328420) was used as the internal control to normalize all data. Three independent biological replicates used in miRNA validation were followed.

### Analysis of statistical data

The statistical analysis of qRT–PCR data was performed using the Graph Pad Prism 5 software (http://www.graphpad.com/scientific-software/prism/). The experimental data values were the mean values from three independent biological replicates and the results are presented as means ±SE, based on three replications. Furthermore, the differences among means were analysed by Bonferroni post-tests. The statistical significance at *P*≤0.05 was also calculated.

## Results

### Iron and zinc contents in different tissues at seed maturation stages

**Fig. 1.**
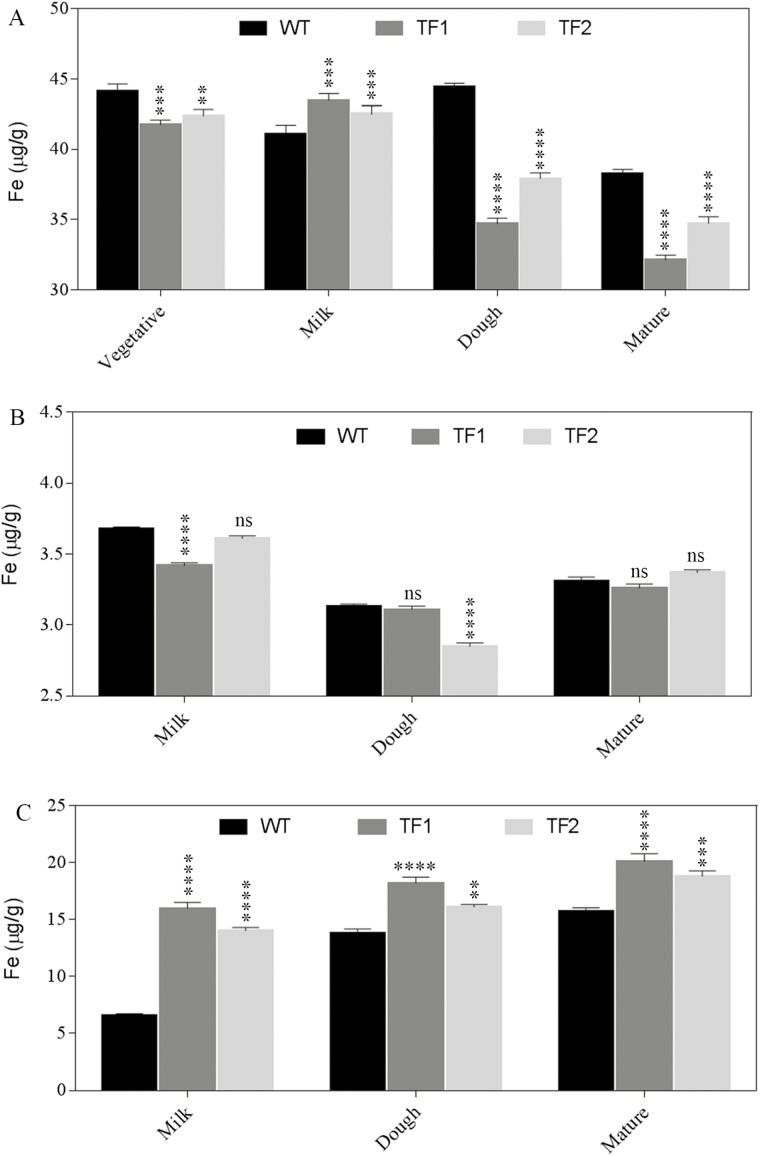
Iron concentration in roots, flag leaves, and seeds of WT and TF rice plants collected during three different seed developmental stages, namely milky, dough, and mature stages. Iron concentration in (A) roots of WT, TF1, and TF2 plants during vegetative, milk, dough, and mature stages of seed development. (B) The iron concentration in flag leaves of TF1 and TF2 plants were more or less similar to that of the WT. (C) Seeds of TF1 and TF2 plants exhibited an elevated amount of iron throughout the three seed developmental stages; the highest iron accumulation is observed during the milk stage. Mean values ±SE are shown. Three biological replicates have been considered. Asterisks indicate significant differences in iron content between the WT and two TF plants (****P<0.0001; ***P<0.001; **P<0.005; ns, non-significant diffference).

**Fig. 2.**
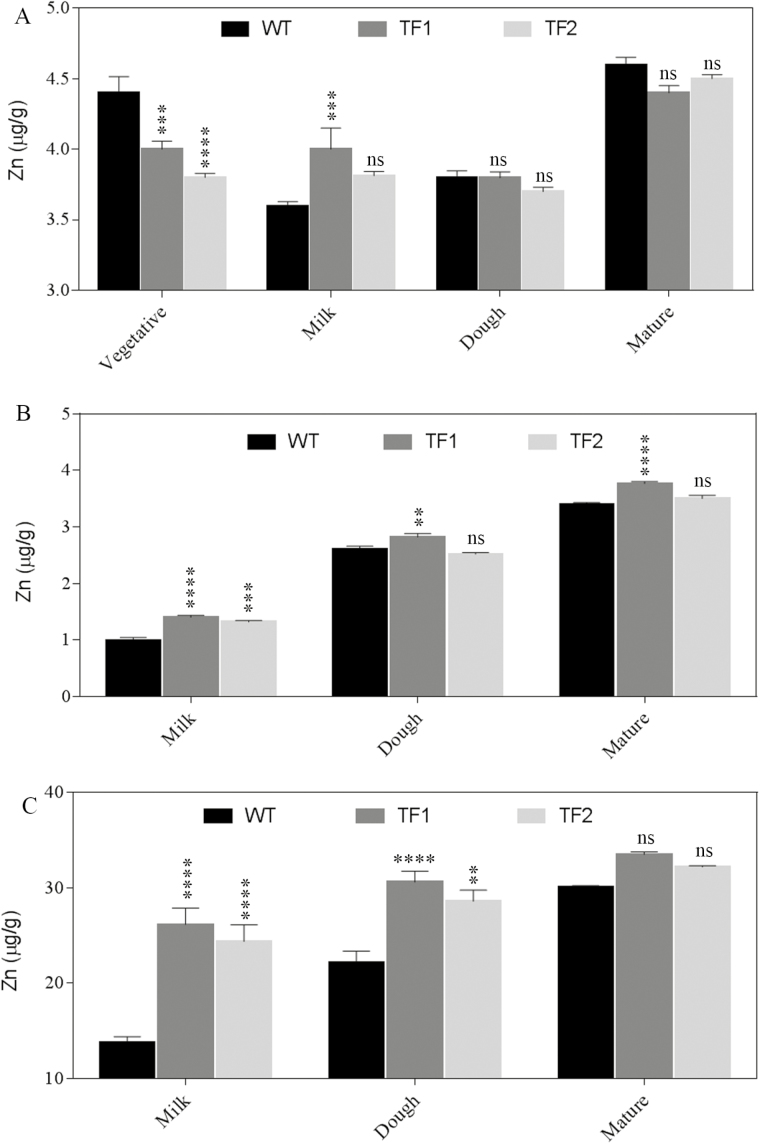
Zinc concentration in roots, flag leaves, and seeds of WT and TF rice plants collected during three different seed developmental stages, namely milky, dough, and mature stages. In roots (A) and in flag leaves (B) of TF plants, the amount of zinc is comparable with that in the WT plants. (C) Seeds of TF plants exhibit higher zinc accumulation throughout the three different stages. Mean values ±SE are shown. Three biological replicates have been considered. Asterisks indicate significant differences in zinc content between the WT and two TF plants (****P<0.0001; ***P<0.001; **P<0.005; ns, non-significant difference).

To identify the most important stage of iron and zinc transport during grain maturation, roots, flag leaves, and immature or mature seeds of WT and TF plants collected during three different grain maturation stages, namely milk, dough, and mature stages, and concentrations of iron and zinc were analysed. Since soil is the primary source of metal ions, roots are considered as the major tissues for iron and zinc transport. The soil was found to be alkaline (pH 8.4) and to contain 64.72mg kg^–1^ and 84.08mg kg^–1^ of iron and zinc, respectively, as the quantities adequate for the paddy field soil (Supplementary Table S3). During the vegetative stage, the iron and zinc content in roots of TF1 and TF2 plants was found to be slightly lower than in those of the WT (Figs 1, 2; Supplementary Table S4). In contrast, the amount of iron and zinc in TF1 and TF2 roots (43.49 µg g^–1^ and 43.44 µg g^–1^ of iron, and 4.00 µg g^–1^ and 3.8 µg g^–1^ of zinc, respectively) was found to be higher compared with WT plants during the milk stage. A similar trend was observed in flag leaves. Interestingly, the immature TF1 and TF2 seeds collected at the milk stage had 15.99 µg g^–1^ and 14.02 µg g^–1^ of iron and 26.16 µg g^–1^ and 24.38 µg g^–1^ of zinc, respectively, which correspond to a rise of 2.13- to 2.43-fold and 1.76- to 1.89-fold for the iron and zinc content, respectively, compared with WT immature seeds. At the milk stage, the iron and zinc accumulation in roots, flag leaves, and immature seeds of the TF2 line was found to be slightly lower than in TF1 but significantly higher than that of the corresponding WT (*P*<0.05). The iron content in roots of TF1 and TF2 plants (34.69 µg g^–1^ and 37.93 µg g^–1^) at the dough stage was found to be slightly lower than that of the WT (44.46 µg g^–1^). In addition, the zinc content was found to be higher in roots of transgenic plants compared with the WT. Iron and zinc contents of flag leaves at all three stages did not exhibit any significant differences between transgenic and WT plants. The differences between iron accumulation in roots of WT and transgenic plants (TF1 and TF2) remained constant during later stages of seed development. The iron and zinc contents in mature TF1 and TF2 seeds remained elevated compared with the WT (iron, 20.10 µg g^–1^ and 18.82 µg g^–1^ in TF1 and TF2, respectively, versus 15.7 µg g^–1^ in the WT; zinc, 33.50 µg g^–1^ and 32.20 µg g^–1^ in TF1 and TF2, respectively, versus 30.1 µg g^–1^ in WT seeds).

### Analysis of sequencing data of the small RNA libraries isolated from WT and TF plants

**Fig. 3.**
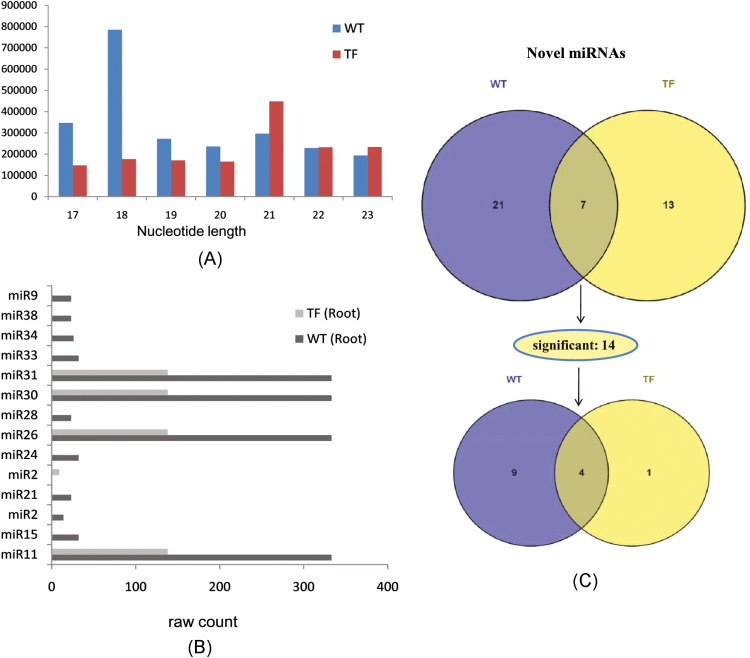
Abundance of small RNA sequences, expression profile of novel miRNAs identified in rice roots, and their distribution in WT and TF roots. (A) Total abundance of small RNA sequences according to the different sizes. In TF roots, 21–23 nucleotide long sequences are more prevalent. (B) Expression profile of significant and novel miRNAs in WT and TF roots (sequence reads). Four novel miRNAs, miR31, miR30, miR26, and miR11, showed higher abundance in WT roots compared with TF roots. (C) Venn diagram of identified novel miRNAs. Among 14 significantly differentially expressed miRNAs, nine belong to WT plants, one is found to be restricted to TF plants, and the remaining four are in common. (This figure is available in colour at *JXB* online.)

Due to the higher iron accumulation observed during the milk stage in roots of TF1 plants compared with those of TF2, roots collected from WT and TF1 plants at this stage of seed development were used for the preparation of small RNA libraries and identification of miRNAs. Sequencing of the WT and TF1 root libraries generated a total of 9 889 255 (57% GC) and 14 711 627 (55% GC) sequence reads, respectively. The sequence length of 49bp was found in both tissues. After removal of low quality sequence reads and adaptor contaminants, distinct sequences obtained were perfectly matched with the rice genome. The differences in size compared with the total small RNA population were determined on the basis of total abundance reads. Sequences abundant in WT roots were 17–19 nucleotides long, whereas they were 21–23 nucleotides long in TF1 roots. However, the prevalence of 21–23 nucleotide long sequences in both tissues was observed (Fig. 3A).

### Identification of differentially expressed conserved and novel miRNA families

A sequence similarity search of specific 21–23 nucleotide long clear sequences obtained from both libraries was conducted using miRPlant, version 3.0 (An *et al.*, 2014). The search allowed us to identify a total of 153 known conserved and non-conserved miRNAs representing 51 families (Supplementary Fig. S1). Of the 153 known miRNAs, 59 were found to be significantly expressed. The statistical analysis of differentially expressed miRNAs was performed with the help of edge R software (http://www.bioconductor.org/packages/release/bioc/html/edgeR.html). The log-fold change (logFC) and log-count per million (logCPM) for each miRNA were calculated and compared between WT and transgenic plants. A *P*-value of ≤0.05 was considered as statistically significant (Supplementary Table S5). Eighteen known miRNAs were found to be conserved in different species, while another six were unique (Table 1). Twelve miRNA families have been designated to be metal responsive in plants (Waters *et al.*, 2012). In our investigation, miR164, miR399, and miR408 were detected which are essential for iron and zinc homeostasis. The roots of TF1 and WT plants were found to contain 29 miRNAs and four miRNAs, respectively. Of the 153 miRNAs, 26 were differentially expressed (Supplementary Fig. S1). The differences in abundance were determined on the basis of sequence reads.

**Table 1.** Various known and significantly expressed miRNA families differentially regulated in roots of TF plants and their relationship to metal homeostasis in plants

Known miRNAs	miRNA families	Nature of miRNAs	Relationship to metal transport
osa_miR156b.1	miR156	Conserved	P, N, S, and Mn (Paul *et al.*, 2015)
osa_miR156c.1			
osa_miR156f.1			
osa_miR156g.1			
osa_miR156h.1			
osa_miR156l			
osa_miR156l.1			
osa_miR160a	miR160	Conserved	P, N, and S (Paul *et al.*, 2015)
osa_miR160b			
osa_miR160c			
osa_miR160d			
osa_miR160e			
osa_miR162b	miR162	Conserved	Cadmium tolerance (Mendoza-soto *et al.*, 2012)
osa_miR164e	**miR164**	Conserved	P, N, S, Mn, and Fe (Paul *et al.*, 2015)
osa_miR166c	**miR166**	Conserved	P, N, and Zn (Paul *et al.*, 2015)
osa_miR166d.1			
osa_miR166h			
osa_miR166i			
osa_miR166j			
osa_miR169c	miR169	Conserved	N and Mn (Paul *et al.*, 2015)
osa_miR169g			
osa_miR169h			
osa_miR169j			
osa_miR169k			
osa_miR169l			
osa_miR169m			
osa_miR171b	miR171	Conserved	N and Zn (Paul *et al.*, 2015)
osa_miR171c			
osa_miR171d			
osa_miR171e			
osa_miR171f			
osa_miR399a	**miR399**	Conserved	P, N, S, Fe, and Zn (Paul *et al.*, 2015)
osa_miR399b			
osa_miR399c			
osa_miR399d			
osa_miR399i			
osa_miR408.1	**miR408**	Conserved	P, N, Cu, and Fe (Paul *et al.*, 2015)
osa_miR531a	miR531	Conserved	Not reported
osa_miR531b			
osa_miR531c			
osa_miR820a	miR820	Unique	Not reported
osa_miR820b			
osa_miR820c			
osa_miR1861e	miR1861	Conserved	Arsenic (Gielen *et al.*, 2012)
osa_miR1861k			
osa_miR1861m			
**osa_miR1862e**	miR1862	Conserved	Not reported
**osa_miR1876**	miR1876	Unique	Nitrogen sensing (Nischal *et al.*, 2012)
**osa_miR1878**	miR1878	Conserved	Not reported
**osa_miR2863b**	miR2863	Conserved	Not reported
**osa_miR2871a**	miR2871	Unique	Not reported
osa_miR3979	miR3979	Unique	Arsenic (Gielen *et al.*, 2012)
osa_miR3979.1			
**osa_miR5072**	miR5072	Conserved	Not reported
**osa_miR5144**	miR5144	Conserved	Not reported
**osa_miR5504**	miR5504	Unique	Not reported
**osa_miR5508**	miR5508	Unique	Not reported
**osa_miR6248**	miR6248	Conserved	Not reported

**Fig. 4.**
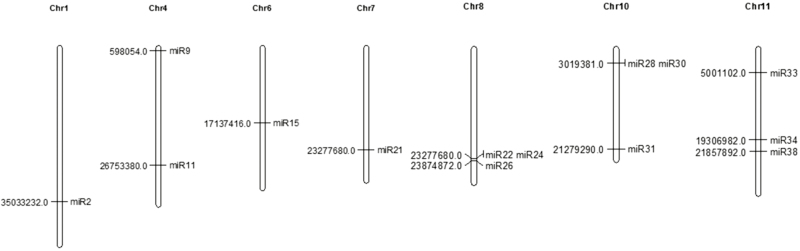
Distribution of novel miR precursor genes in chromosomes.The position of each miRNA has been marked. miR2 on chromosome 1, miR9 and miR11 on chromosome 4, miR15 on chromosome 6, miR21 on chromosome 7, miR22, miR24, and miR26 on chromosome 8, miR30 and miR31 on chromosome 10, and miR33, miR34, and miR38 on chromosome 11.

A total of 41 novel miRNAs were found in the roots of both WT and transgenic lines (Supplementary Table S6). The novel miRNAs were identified following computational and experimental data that include excision of the mature miRNA sequences from the precursor stem–loop structure and the *dcl*-dependent structure, and sequencing of miRNA–miRNA* (in the absence of the *dcl* mutant) (Meyers *et al.*, 2008). In the present study, the rice genome was scanned for stem–loop hairpin structures and the sequences that showed perfect homology with the small RNA sequences from the library were analysed for the stem–loop hairpin structure. The genomic sequences producing the successful hairpin precursor structures and fulfilling the criterion of an miRNA precursor were selected for further analysis. A total of 917 800 and 1 063 956 novel sequences and 545 and 1020 novel miRNAs were found in WT and TF1 roots, respectively. Based on a score of ≥3.5, a total of 28 and 20 putative novel miRNAs were selected from the WT and TF1 library. Among 41 putative novel miRNAs identified, 14 were significantly expressed (*P*≤0.05). Interestingly, four of the 14 novel miRNAs—miR11, miR26, miR30, and miR31—were found to be differentially expressed in both types of roots, while 10 miRNAs were found to be unique (Fig. 3B). Nine unique novel miRNAs, miR15, miR2, miR21, miR24, miR28, miR33, miR34, miR38, and miR9, were expressed in WT roots, while only miR2 was expressed in TF1 roots (Fig. 3B, C). Moreover, the sequences of several differentially expressed matured novel miRNAs common in WT and TF1 roots, miR11, miR26, miR30, and miR31, miR15, miR24, and miR33, and miR21, miR28, miR38, and miR9, were found to be identical. However, they were identified as different as they originated from different *MIR* genes present on different chromosomal loci (Supplementary Table S7; Supplementary Fig. S2). This suggests that several identical novel miRNAs originated by duplication of sequences that are localized on different chromosomes. The chromosomal mapping of 14 novel miRNAs was performed using Mapchart software (Fig. 4). Of the 14 novel miRNAs, two linkage groups have two miRNAs each while the rest of the linkage groups contained a single miRNA. The miRNAs miR22 and miR24 belong to one linkage group; and miR28 and miR30 can be considered as belonging to different linkage groups. There were no homologies identified for all 14 significant novel miRNAs in other plant species, indicating their rice-specific nature. The sequence of the four differentially expressed novel miRNAs, miR11, miR26, miR30, and miR31, were of 333 sequence reads in WT and 138 sequence reads in TF1 roots. These four novel miRNAs were found to be reduced in roots of TF1 plants - during the milk stage of seed development.

### Annotation of novel miRNAs by target gene identification

**Fig. 5.**
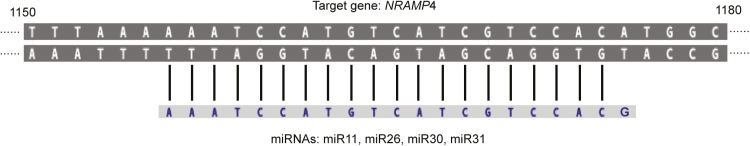
Sequence complementary matches between novel miRNAs and their target *NRAMP*4 gene. Perfect complementarity of four novel miRNAs (20 of 21 nucleotides) with the *NRAMP*4 gene (from 1156 to 1176 of chromosome 1) indicates *NRAMP*4 as their corresponding target gene.

To establish a relationship with potent iron transporters, the prediction of the target gene of the novel miRNAs is crucial, which is performed using the computational algorithm software psRobot, which has maximum efficiency (Srivastava *et al.*, 2014). In psRobot, the highest cut-off score of 2.5 was selected for target gene prediction. It was predicted that 99 mRNAs were the target of 14 miRNAs and 90% of predicted target transcripts were found to be normalized by cleavage, thereby inhibiting their translation (Table 2). The number of predicted target genes for miRNAs varied from one to 32. The lowest score indicates a specific targeted transcript. The chromosomal locus and Gene Ontology (GO) of the target gene were analysed using the Rice Genome Annotation Project 7 database. The predicted targets of novel miRNAs were found to encode a wide range of proteins such as a peptide transporter, DNA invertase/pectin methyl esterase, a retrotransposon, and *S*-adenosylmethionine (SAM) methyl esterase. The DNA invertase/pectin methyl esterase (LOC_Os08g01670.1) was found to be a common target of miR15, miR24, and miR33. Of note, the transcript encoding a metal transporter NRAMP4 was predicted as a target of four differentially expressed novel miRNAs, miR11, miR26, miR30, and miR31. The role of NRAMP4 in iron and zinc transport is well established in plants (Oomen *et al.*, 2009). The sequence analysis showed the perfect complementary match for 20 nucleotides of each of the four novel miRNAs (21 nucleotides in length) and the *NRAMP*4 gene (from 1156 to 1176 of chromosome 1; mRNA positions 17 454 023–17 465 049) as depicted in Fig. 5. This finding confirmed the role of the four novel miRNAs (miR11, miR26, miR30, and miR31) in iron transport.

**Table 2.** Different putative novel miRNAs significantly expressed in roots of WT and TF1 rice plants and their predicted target genes

miR ID	Sequence (5'–3')	No. of nucleotides	WT (read)	TF (read)	Target gene locus ID	Annotation	Score	Total target genes
**miR11,** **miR30,** **miR31,** **miR26**	AAATCCATGTCATCGTCCACG	21	333	138	LOC_Os01g31870.8	DNA natural resistance-associated macrophage protein (NRAMP4), putative, expressed, metal transport	0.2	20
**miR15, miR33, miR24**	ACCTGCGGGTCTTCGGCTGCC	21	32	0	LOC_Os08g01670.1	DNA invertase/pectin methyl esterase inhibitor family protein, putative, expressed	1.5	7
**miR2**	GCCGCGTCGTCGTCGTCG	18	14	0	LOC_Os10g02340.1	DNA peptide transporter PTR2, putative, expressed	0.0	10
**miR22**	TTCAGTTTCCTCTAATATCTCG	22	0	9	LOC_Os04g05710.1	cDNA retrotransposon protein, putative, Ty3-gypsy subclass	2.5	10
**miR21,** **miR28, miR38,** **miR9**	GCCTTGATCGCTATTGACC	19	23	0	LOC_Os01g50610.1	cDNA SAM-dependent carboxyl methyl transferase	2.0	32
**miR34**	TAACACTTCCGTCAATTCTTG	21	26	0	LOC_Os03g31460.1	cDNA formin-like protein 20	1.2	6

### Differential expression analysis of novel and known miRNAs

**Fig. 6.**
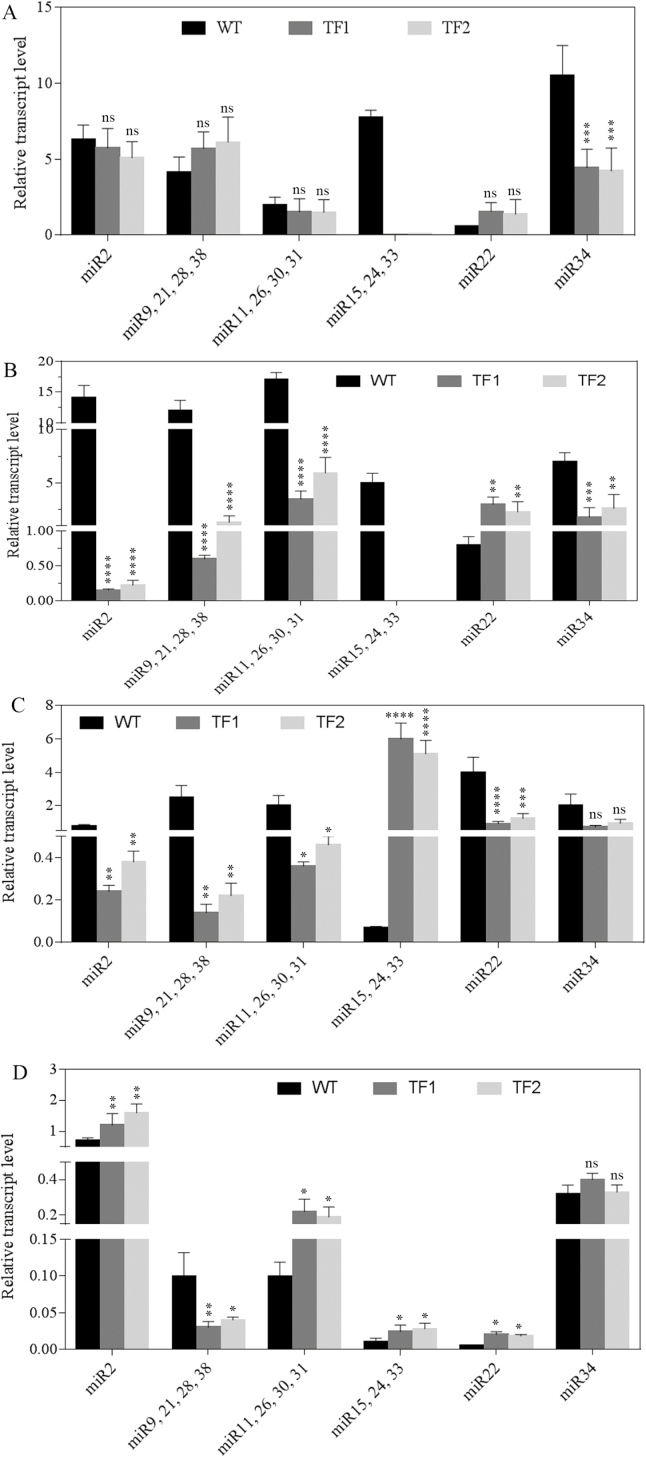
qRT–PCR-mediated validation of novel miR precursor RNAs in WT, TF1, and TF2 plants during the vegetative phase and milk stage of seed development. The expression graph includes the relative transcript level of novel miR precursor RNAs in (A) roots collected during the vegetative stage, (B) roots collected during the milk stage, (C) flag leaves collected during the milk stage, and (D) seeds collected during the milk stage. miR11, miR26, miR30, and miR31 showed a significant reduction in roots of TF1 and TF2 plants compared with WT plants. Mean values ±SE are shown (*n*=3 biological replicates). Asterisks indicate significant differences in the relative transcript level of miR precursor RNAs between the WT and two TF plants (****P<0.0001; ***P<0.001; **P<0.005; *P<0.05; ns, non-significant diffference).

**Fig. 7.**
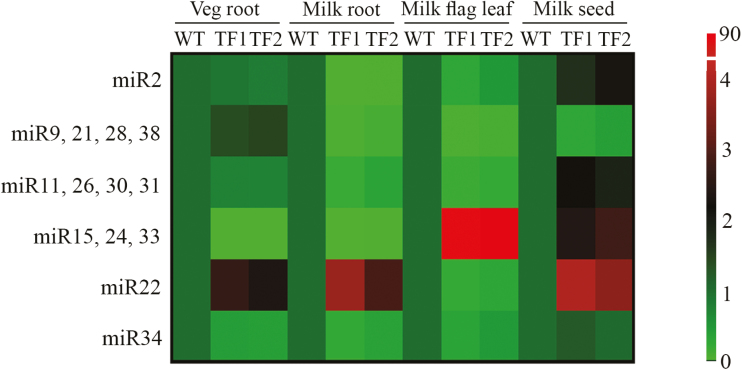
Heat map showing comparative fold change values of novel miR precursor RNAs between WT and TF plants in different tissues. The scale represents fold change values. (This figure is available in colour at *JXB* online.)

To validate the expression profile, the expression of the novel miRNAs was analysed by qRT–PCR in roots, leaves, and immature seeds during the milk stage of seed development. In the vegetative stage, most of the novel miRNAs were differentially regulated in TF1 and TF2 roots. The relative transcript levels of four miRNAs, namely miR11, miR26, miR30, and miR31, were found to be slightly down-regulated in the transgenic lines (0.76- and 0.75-fold in TF1 and TF2 roots) (Figs 6, 7; Supplementary Table S8). Most of the novel miRNAs except miR22 were found to be considerably down-regulated in both TF1 and TF2 roots compared with the WT during the milk stage. Interestingly, the transcript levels of the four miRNAs (miR11, miR26, miR30, and miR31) were found to be 0.20- and 0.34-fold lower in TF1 and TF2 roots as compared with WT roots during the milk stage. On the other hand, miR15, miR24, and miR33 showed 85.71- and 72.85-fold higher expression in flag leaves of TF1 and TF2 plants than those of WT plants. Other novel miRNAs were found to be down-regulated in flag leaves of the transgenic lines. In addition, the expression of all the significant novel miRNAs was found to be reduced in the developing seeds of both WT and transgenic lines.

Since large numbers of known miRNAs were identified, 10 known miRNAs were selected for validation of MIR gene expression based on their role in transport of iron or other metals in various plant species. The comparison of changes in miRNA expression between WT and transgenic lines (TF1 and TF2 plants) was found to be in line with the sequence analysis (Supplementary Fig. S3).

### Validation of target gene expression

**Fig. 8.**
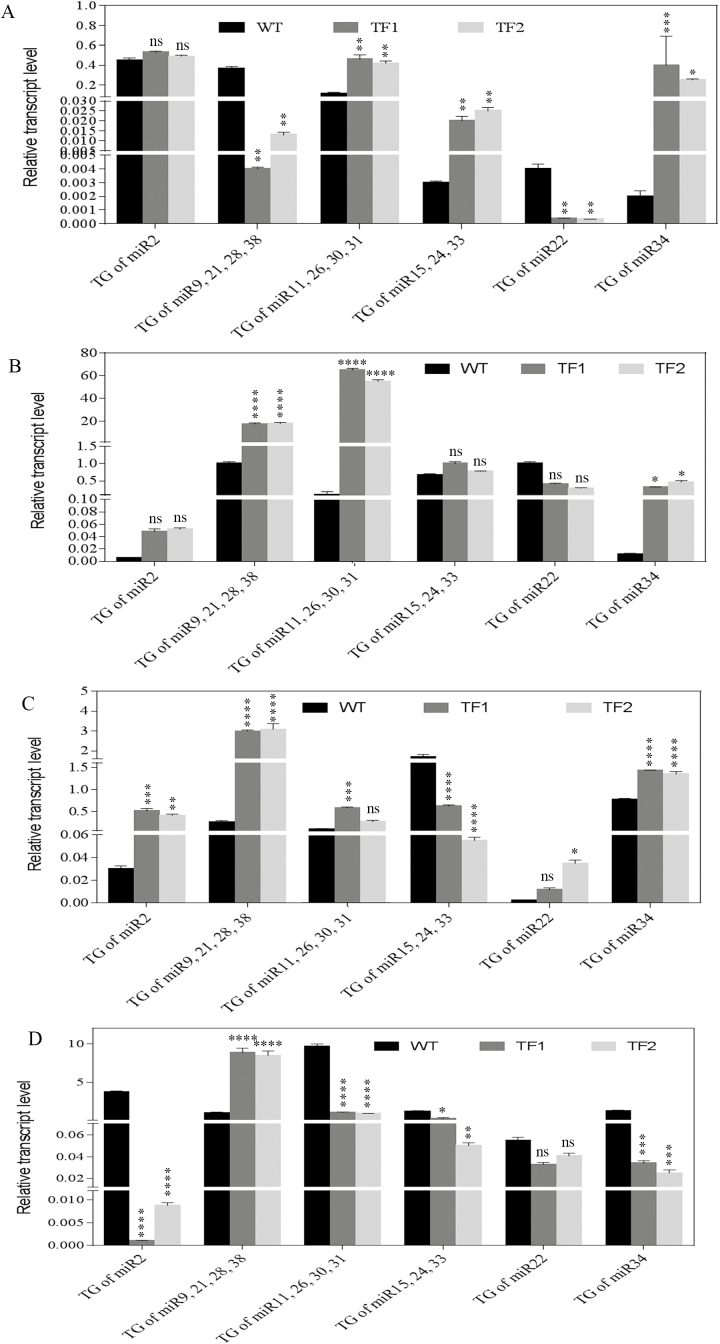
The relative mRNA expression profile of target genes of novel miRNAs showing their relative transcript level in (A) roots collected during the vegetative stage, (B) roots collected during the milk stage, (C) flag leaves collected during the milk stage, and (D) seeds during the milk stage. Mean values ±SE are shown (*n*=3 biological replicates). Asterisks indicate significant differences in the relative transcript level of miR target genes between the WT and the two TF plants (****P<0.0001; ***P<0.001; **P<0.005; *P<0.05; ns, non-significant diffference).

The expression of the target genes of corresponding novel and significant miRNAs was found to be altered in different tissues of transgenic rice plants. Differences in corresponding mRNA levels of predicted target genes were validated by qRT–PCR. The relative mRNA expression for all predicted target genes was altered in the transgenic lines compared with WT plants (Fig. 8; Supplementary Table S9). The expression of miRNAs and their target genes was found to be inversely correlated in most cases. For example, the expression of the SAM-dependent carboxyl methyl transferase gene (LOC_Os01g50610.1), the target gene of four novel miRNAs, namely miR9, miR38, miR21, and miR28, was found to be 17.42- and 17.86-fold higher in TF1 and TF2 roots during the milk stage of development, which was correlated with its lower miRNA expression (0.05- and 0.11-fold down-regulation). In addition, during the milk stage, the relative transcript level of *OsNRAMP*4 (LOC_Os01g31870.8) was elevated from 0.107 in WT roots to 65.24 and 55.39 in TF1 and TF2 roots, respectively. Also, in TF1 and TF2, the expression level of *NRAMP*4 increased significantly in the milk stage in comparison with the vegetative stage, while it remained almost unchanged in the WT. The four novel miRNAs (miR11, miR26, miR30, and miR31) may have some role in the alteration of their target *OsNRAMP*4 gene expression. In addition, the relatively higher abundance of these novel miRNAs in WT roots compared with TF1 and TF2 plants presumably minimizes the *NRAMP*4 gene expression. In flag leaves, the decreased level of *NRAMP*4 was reported in both WT and transgenic lines. However, there was no major difference identified in the expression of *NRAMP*4 between the WT and TF lines, and hence in iron transport in leaves. Moreover, the expression of *NRAMP*4 in TF1 and TF2 immature seeds was significantly down-regulated by 0.10-fold compared with WT seeds, suggesting a lower iron transport by seeds than by roots. The expression of the rest of the target genes in roots, flag leaves, and immature seeds also followed a similar pattern of gene regulation.

### Differential expression of various transporters during seed maturation stages

**Fig. 9.**
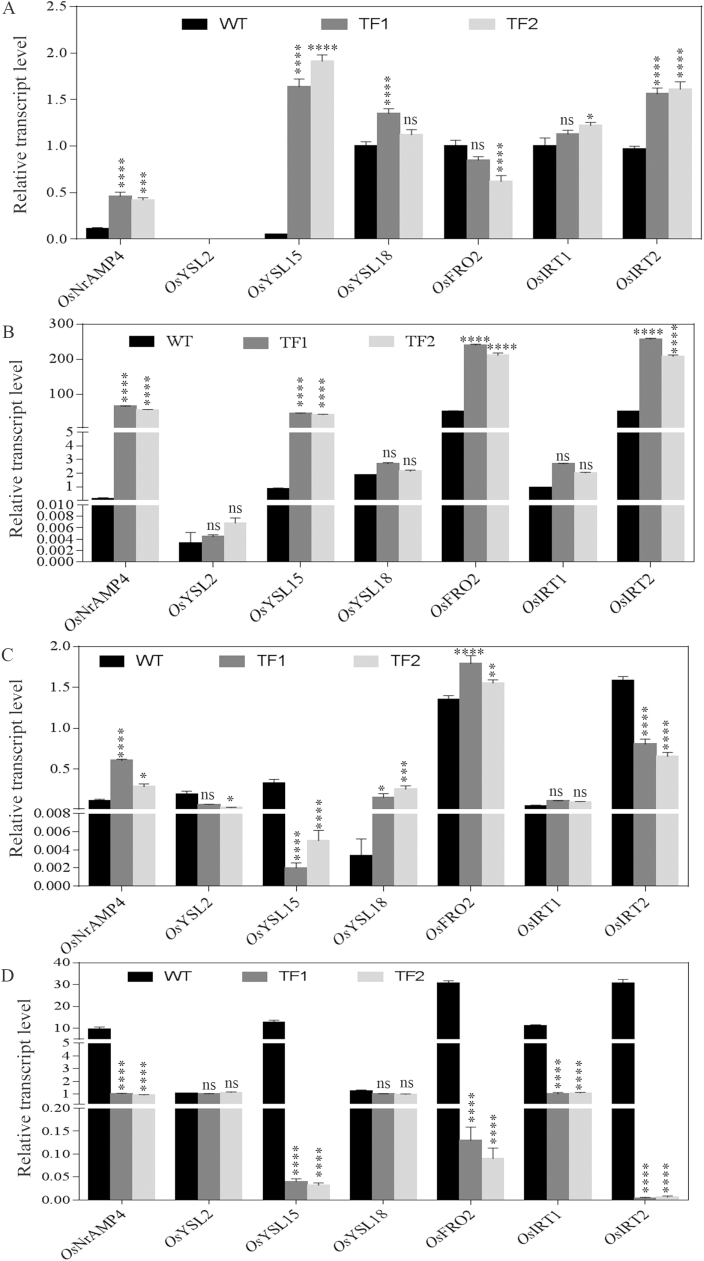
Expression profile of various transporter genes in WT and TF1 and TF2 plants showing the relative transcript level of the respective mRNAs in (A) roots collected during the vegetative stage, (B) roots collected during the milk stage, (C) flag leaves collected during the milk stage, and (D) seeds during the milk stage. Mean values ±SE are shown (*n*=3 biological replicates). Asterisks indicate significant differences in the relative transcript level of transporter genes between WT and the two TF plants (****P<0.0001; ***P<0.001; **P<0.005; *P<0.05; ns, non-significant diffference).

**Fig. 10.**
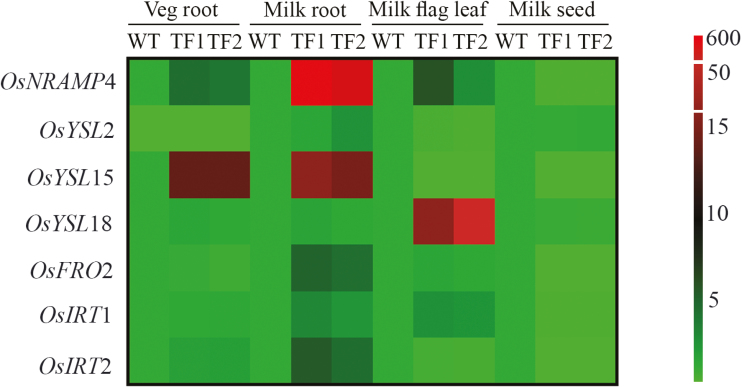
Heat map showing a comparison of the relative expression of transporter genes between WT and TF1 and TF2 plants. The scale represents fold change values. *OsNRAMP*4, *OsYSL*15, *OsFRO*2, and *OsIRT*2 showed higher abundance in roots of TF1 and TF2 plants during the milk stage, whereas *OsNRAMP*4, *OsYSL*15, *OsFRO*2, *OsIRT*1, and *OsIRT*2 are found to be more abundant in WT seeds. (This figure is available in colour at *JXB* online.)

To investigate the role of other transporters in different tissues during the milk stage, the expression levels of seven transporters including NRAMP4 was analysed by qRT–PCR analysis. During the milk stage of seed development, the *NRAMP*4 transcript was significantly enhanced in both TF roots and in WT seeds. The role of different metal transporters, such as YSL2, YSL15, YSL18, FRO2, IRT1, and IRT2, has been established for iron homeostasis in rice plants (Kobayashi *et al.*, 2014). Intriguingly, during the vegetative stage, transporters such as NRAMP4, YSL15, and IRT2 were up-regulated in transgenic roots compared with WT roots (Figs 9, 10; Supplementary Table S10).The expression of *YSL*2 was undetected in both lines during the vegetative stage. The differences in the expression profile of transporters were found to be more pronounced once the plant growth advanced to the milk stage. During the milk stage, in both TF roots *OsYSL*15, *OsFRO*2, and *OsIRT*2 transcripts along with *OsNRAMP*4 were found to be expressed notably more than in WT roots. The relative transcript level of *OsYSL*15 and *OsFRO*2 was significantly increased from 0.86 to 45 and from 51.53 to 239.69, respectively, in the TF1 roots. The highest relative transcript level was found to be of *OsIRT*2, as indicated by levels of 256.81 and 50.85 in TF1 and WT roots, respectively. However, no such difference was observed in expression of transporters, except *YSL*18, in flag leaves of both WT and TF plants. In addition to *OsNRAMP*4, the relative transcript levels of *OsYSL*15, *OsFRO*2, *OsIRT*1, and *OsIRT*2 were found to be enhanced in immature seeds of WT compared with TF plants. The maximum relative transcript level of 30.84 and 30.75 was recorded for *OsFRO*2 and *OsIRT*2 transporters, respectively, in WT immature seeds. Among the transporters, *OsFRO*2 and *OsIRT*2 showed a major role in iron transport in developing WT seeds, whereas *OsIRT*2 and *OsFRO*2 were found to be predominant in roots of TF plants during the milk stage of seed development.

## Discussion

In the last decade, the development of high iron rice by biotechnological approaches is a significant milestone in crop improvement programmes (Masuda *et al.*, 2013). Based on the transgene used, the iron content of consumable milled seeds varies. The seed-specific overexpression of iron storage protein OsFER2 (endogenous ferritin) has been shown to increase the iron content of transgenic seeds compared with the WT (Paul *et al.*, 2012). Furthermore, a few transporters were also used as transgenes to mobilize iron in plants (Kobayashi *et al.*, 2014). The combination of both strategies was very effective in loading of iron in seeds. Recently, Wang *et al.* (2013) produced an in-depth insight into iron transporters by overexpression of *NAS* and *PvuFER*1 genes. Here, we dissected the role of various transporters in soy*FER*1-overexpressing iron-rich transgenic rice grains and their regulation by a novel group of miRNAs during seed developmental stages.

A number of plant transporters are activated during different seed developmental stages to maintain nutrient homeostasis (Chu *et al.*, 2010). In the present study, the most effective and significant differences for iron and zinc accumulation were observed in seeds during different seed development stages. This was in line with a previous study in which cereals including rice grain store nutrient minerals during three different seed developmental stages—milk, dough, and mature (Lu *et al.*, 2013). We noticed an increase in iron and zinc accumulation from the milk stage to the mature stage. However, the major difference in iron content between transgenic plants and their WT counterpart was recorded during the milk stage, suggesting the up-regulation of some transporters in roots of TF plants. In an earlier study, iron transporters such as OsIRT1 and OsYSL2 were found to be up-regulated in transgenic roots during the milk and dough stages (Wang *et al*., 2103). The expression of transporters alters with the iron content of the soil. The results of this study showed that during the milk stage of development, some transporters were significantly up-regulated in TF roots compared with WT plants. This is to fulfil the higher demand of iron in transgenic seeds compared with WT plants and thus maintain iron homeostasis.

MiRNAs play a vital role in maintaining nutrient homeostasis in plants by regulating the expression of diverse genes. Interestingly, the iron-responsive miRNA members belonging to the miR399 and miR408 families were found to be significantly expressed in TF roots. A couple of miRNA families such as miR399 and miR408 are widely distributed among plants and contribute nutrient homeostasis including that of iron and zinc (Waters *et al.*, 2012). The amplification of these miRNAs observed in TF plants might facilitate the expression of iron transporters by minimizing the activity of a repressor gene. The detailed investigation of this signalling cascade can provide a novel mechanism of iron homeostasis. Another family of miRNAs, miR166, was found to be up-regulated in TF roots and is known to play a role in zinc homeostasis in *Sorghum bicolor* (Li *et al.*, 2013). In addition, four of the 14 significantly expressed putative novel miRNAs—miR11, miR26, miR30, and miR31—that target the metal transporter gene *OsNRAMP*4 were found to be significantly down-regulated in TF roots. Albeit that OsNRAMP4 has not been functionally characterized so far, the role of NRAMP4 in iron transport has been investigated in several plant species (Lanquar *et al.*, 2005; Oomen *et al.*, 2009). In addition, the role of other NRAMP molecules such as OsNRAMP5 has been established in iron transport in rice (Ishimaru *et al.*, 2012). Blast analysis (in the Rice genome annotation project) of our *OsNRAMP*4 gene sequence suggested its homology with different NRAMP genes such as *NRAMP*1, *NRAMP*5, and *NRAMP*6. Also, GO analysis (biological process) revealed an iron transport role for *OsNRAMP*4. A domain search for the protein showed the presence of the SLC5-6-like sbd superfamily domain in the protein which specifies transporter activity. Interpro analysis also indicates that our protein belongs to the NRAMP protein family (IPR001046), and a divalent cation transporter role has been predicted. However, functional characterization of OsNRAMP4 needs to be investigated in order to confirm its iron transport activity or crosstalk with other divalent cations, if any.

**Fig. 11.**
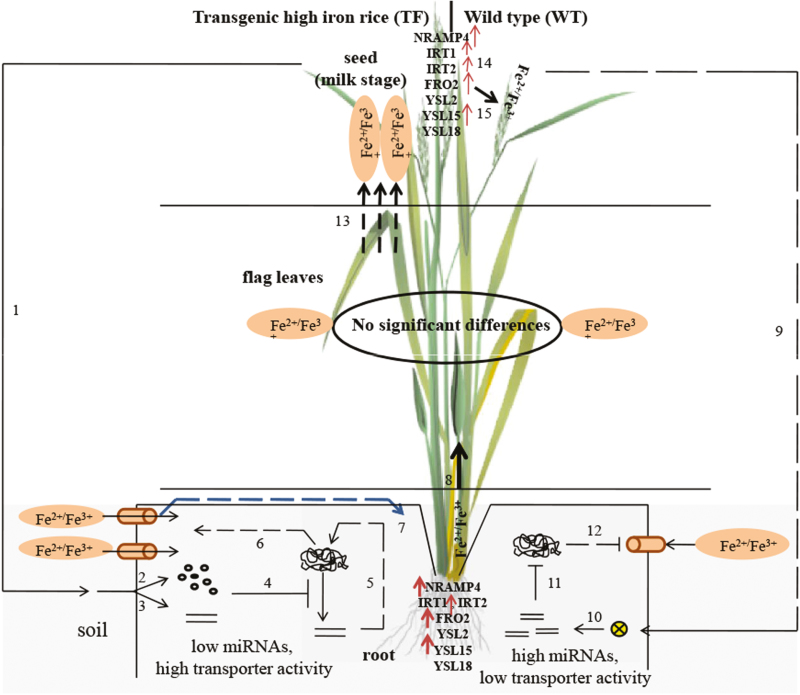
Postulated signalling pathway of miRNA-mediated regulation of iron transport in transgenic rice plants. In TF plants, (1) during the milk stage of seed development, the higher abundance of iron in seeds induces signalling molecules (unknown) in roots, The signalling molecules may be (2) protein molecules (transcription factors) or (3) small RNAs which act as anti-miRNAs. (4) Signalling molecules in turn down-regulate four novel root-specific miRNAs, miR11, miR26, miR30, and miR31. (5) A lower abundance of miRNAs activates the transcription of the target transporter, NRAMP4, and (6) ultimately higher expression in roots; (7) a higher abundance of *NRAMP*4 along with other transporters such as *OsYSL*15, *OsFRO*2, and *OsIRT*2 facilitates iron transport through TF roots. (8) High iron (ferrous/ferric ion) is transported to the shoot from the roots. In contrast, (9) the iron concentration in WT seeds suppresses the formation of signalling molecules in roots, which in turn (10) activate accumulation of the four novel miRNAs in roots. (11) Elevated miRNAs inhibit *NRAMP*4 gene expression and (12) finally reduced transporter accumulation in WT roots. (13) Elevated amounts of iron are transported to seeds from flag leaves where no significant differences in transporter gene activation is recorded. (14) In WT seeds, *OsNRAMP*4, *OsYSL*15, *OsFRO*2, *OsIRT*1, and *OsIRT*2 are activated for (15) iron loading in grain that helps to maintain iron homeostasis. The arrows indicates activation of transporters, increased state of different transporters, and the step by step signal transduction mechanism. (This figure is available in colour at *JXB* online.)

The miRNA expression validated by qRT–PCR supported the expression profile of novel miRNAs analysed by next-generation sequencing (NGS). During the milk stage of seed development, the increased expression of miR11, miR26, miR30, and miR31 might have inhibited *NRAMP*4 expression. In contrast, the reduced amount of these novel miRNAs in TF roots can play a role in the up-regulation of the *NRAMP*4 gene, as evidenced by the relative transcript levels of 65.24 and 55.39 in TF1 and TF2 plants compared with 0.107 in WT plants during the milk stage. This therefore suggests that increased iron requirements in transgenic seeds may initiate a cellular signal which in turn can suppress these novel miRNAs, thereby activating NRAMP4 (Fig. 11).

We measured mRNA expression of various classes of transporters that are activated by high iron conditions. In rice, the role of some essential iron transporter families such as YSL, IRT, and FRO2 has previously been studied (Bughio *et al.*, 2002; Ishimaru *et al.*, 2006, 2007; Inoue *et al.*, 2009; Chandel *et al.*, 2010). Along with NRAMP4, the expression of *OsYSL*15, *OsFRO*2, and *OsIRT*2 was found to be augmented in roots of TF plants that facilitate iron accumulation in seeds during the milk stage. However, in WT plants, the higher amount of iron accumulation in the dough stage compared with the milk stage suggests the activation of transporters in developing seeds during the dough stage. The up-regulation of four iron transporters (OsNRAMP4, OsYSL15, OsFRO2, and OsIRT2) in seeds of WT plants helps to increase the iron accumulation. However, in TF seeds, the level of iron accumulation was found to be much higher throughout all the seed developmental stages. The maximum difference in iron accumulation between TF and WT seeds was observed during the milk stage, and a moderate difference was found at the mature stage. This might be due to endosperm-specific overexpression of ferritin in TF seeds that activates the array of transporters in transgenic roots during the milk stage. In contrast, few transporters such as NRAMP4 and YSL15 were found to be more activated in roots of transgenic plants compared with the WT at the juvenile stage. This may have two explanations. First, the reduced iron content in roots of TF1 and TF2 plants during the vegetative stage is thought to induce the expression of *NRAMP*4 which exaggerates the vacuolar iron export to maintain cellular iron homeostasis. A similar phenomenon was observed in Arabidopsis where *AtNRAMP*3 and *AtNRAMP*4 were found to be up-regulated in seeds during germination (Lanquar *et al.*, 2005). Secondly, a very small amount of ferritin transcript in roots (Supplementary Fig. S4) of transgenic plants may activate the transporters. However, in the future, further investigations are necessary to understand the detailed mechanisms. In TF seeds, zinc accumulation was also significantly higher during the milk stage, indicating that similar transporters are shared by iron and zinc. The role of iron transporters in mobilizing zinc has been studied previously (Ishimaru *et al.*, 2011).

Herein, we reported for the first time the involvement of miRNAs in the regulation of iron transport and unravelled the molecular mechanisms of iron and zinc accumulation in transgenic rice grains. During the milk stage of development, miRNAs such as miR11, miR26, miR30, and miR31 may activate the OsNRAMP4 transporter and thus can regulate iron trafficking in rice. Understanding the molecules involved in activation or inhibition of miRNAs responsible for iron accumulation may help to dissect the detailed mechanism of iron homoeostasis in crop plants.

## Supplementary data

Supplementary data are available at *JXB* online.

Table S1. Primers used for quantitative analysis of novel miRNAs.

Table S2. Primers used for quantitative analysis of target genes and transporters.

Table S3. Soil analysis report showing iron and zinc content.

Table S4. Iron and zinc concentrations of TF1 and TF2 plants.

Table S5. Known miRNAs differentially expressed in roots of rice plants.

Table S6. Novel miRNAs differentially expressed in roots of rice plants.

Table S7. Different chromosomal loci of novel miRNAs,

Table S8. qRT–PCR-mediated validation of expression of novel miRNAs in TF1 and TF2 plants

Table S9. qRT–PCR-mediated validation of expression of target genes in TF1 and TF2 plants.

Table S10. qRT–PCR-mediated validation of expression of transporters in TF1 and TF2 plants.

Figure S1. Venn diagram of known miRNAs.

Figure S2. Precursor sequences of miRNAs.

Figure S3. qRT–PCR-mediated validation of expression of known miRNAs in TF1 and TF2 plants.

Figure S4. qRT–PCR analysis of soy*FER*1 gene expression in shoots of WT, TF1, and TF2 plants during the vegetative stage.

Supplementary Data

## Abbreviations:

DMA: deoxymugineic acid
EBR: epibrassinolide
FRO: ferric chelate reductase oxidase
GO: Gene Ontology
HMA: 3-hydroxymugineic acid
IDA: iron deficiency anaemia
IRT: iron-regulated transporter
MA: mugineic acid
NGS: next-generation sequencing
NRAMP: natural resistance-associated macrophage protein
SAM: *S*-adenosylmethionine
TIGR: The Institute for Genome Research
TF: transgenic ferritin-overexpressing plant;
WT: wild type
YSL: yellow stripe like.
