# Supplementary material for: Analysis of high iron rice lines reveals new miRNAs that target iron transporters in roots
Source: J Exp Bot. 2016 Oct 11;67(19):5811–24. doi: 10.1093/jxb/erw346 (PMC5066498; doi:10.1093/jxb/erw346)
Supplement: Supplementary Data [file supp_67_19_5811__index.html]

Analysis of high iron rice lines reveals new miRNAs that target iron transporters in roots — Analysis of high iron rice lines reveals new miRNAs that target iron transporters in roots — Supplementary Data 

# Analysis of high iron rice lines reveals new miRNAs that target iron transporters in roots

## Supplementary Data

Data files

- supplementary\_tables\_S1\_S10\_figures\_S1\_S4.pdf - Supplementary Data
