# Supplementary material for: Analysis of high iron rice lines reveals new miRNAs that target iron transporters in roots
Source: J Exp Bot. 2016 Oct 11;67(19):5811–24. doi: 10.1093/jxb/erw346 (PMC5066498; doi:10.1093/jxb/erw346)

Supplementary Table S1: Designed miRNA primers of putative novel miRNAs (14 significant) for qRT-PCR analysis. (A) primers for mature miRNAs, (B) Stem-loop RT primer

**Novel miRNAs**	**miRNA specific forward primer**	**Length (bp)**	**Tm**
miR11, miR30, miR31, miR36	5’GCGGAAATCCATGTCATCGA3’	20	60˚C
miR15, miR24, miR33	5’ACCTGCGGGTCTTCGGCA3’	18	60˚C
miR2	5’GGCCGCGTCGTCGTCGAA3’	17	62˚C
miR22	5’GGCGGTTCAGTTTCCTCTAA3’	20	60˚C
miR21, miR28, miR38, miR9	5’CGGGCCTTGATCGCTATTGA3’	20	62˚C
miR34	5’GGCGGTAACACTTCCGTCAA3’	20	62˚C
U6	F:5’ATTTGGACCATTTCTCGATTTGT3’<br>R: 5’TGGAACGATACAGATAAGATTAG3	23<br>23	59˚C

(B)

Novel miRNAs	RT Primer sequence
miR11, miR30, miR31, miR36	GTCGTATCCAGTGCAGGGTCCGAGGTATTCGCACTGGATACGACCGTGGA
miR15, miR24, miR33	GTCGTATCCAGTGCAGGGTCCGAGGTATTCGCACTGGATACGACGGCAGC
miR2	GTCGTATCCAGTGCAGGGTCCGAGGTATTCGCACTGGATACGACCGACGA
miR22	GTCGTATCCAGTGCAGGGTCCGAGGTATTCGCACTGGATACGACCGAGAT
miR21, miR28, miR38, miR9	GTCGTATCCAGTGCAGGGTCCGAGGTATTCGCACTGGATACGACGGTCAA
miR34	GTCGTATCCAGTGCAGGGTCCGAGGTATTCGCACTGGATACGACGGCAGC

Supplementary Table S2: Primer sequences of various target genes and transporters obtained from Primer3 software

Target genes (TGs) of novel miRNAs	Primer sequence
TG of miR11, miR26, miR30, miR31; ***OsNRAMP*4** (LOC_Os01g31870.8)	F: 5'CTTGCACATGTTGGTCCTGG3'<br>R: 5'TTCACCCATGCACAACTCAG3'
TG of miR2; **DNA peptide transporter PTR2, putative, expressed** (LOC_Os10g02340.1)	F: 5'GACTACCGGATCATCGAGCA3'<br>R:5'GAAGAAGAACACCACCGTCG3'
TG of miR22; **cDNA retrotransposon protein, putative, Ty3-gypsy subclass** (LOC_Os04g05710.1)	F: 5'ATTTACCTGGGATGCCACCA3'<br>R: 5'AAGGTGATGAACTGGGACGT3'
TG of miR15, miR24, miR33; DNA **invertase/pectin methylesterase inhibitor family protein**, putative, expressed (LOC_Os08g01670.1)	F: 5'GATGGGGCAGCAGGACTAC3'<br>R: 5'TGTTGGATGCAGGAGAGGAG3'
TG of miR21, miR28, miR38, miR9; cDNA **SAM dependent carboxyl methyltransferase** (LOC_Os01g50610.1)	F: 5'TAGGGGAGATCGAGGAGGAG3'<br>R: 5'CTGAGCATGGACTCCTGGAT3'
TG of miR34; **cDNA formin-like protein 20** (LOC_Os03g31460.1)	F: 5'TGCTGGTGTACATGGAGGAG3'<br>R: 5'AACAGGTCTGAGGATGAGGC3'
**Transporters**	
OsYSL2	F: 5' AGAACACCGTTGTCCAGACC3'<br>R: 5' TAAGGAGCCCAACGAAGCTA3'
OsYSL15	F: 5' TTATCAACGGGTTCCACACA3'<br>R: 5' GTTTCCAGGCTTTCAGACCA3'
OsYSL18	F: 5'TGGCCTTCTTCTTCCTCGTT3'<br>R:5'GAGACTTGCCTCTTCGCTTG3'
OsFRO2	F:5'TCAACTTCTGCAAAAGCCCC3'<br>R:5'TCTCGTACAGCTTCTCCGTC3'
OsIRT1	F: 5'ACCAGATGTTCGAGGGGATG3'<br>R: 5'CTGTTGTCCCTGTACACCCT3'
OsIRT2	F:5' ATCGCGTCATTGTGCAGGT3'<br>R: 5'CGTCGTGGTGGAGAAGAAG3'
ß-tubulin	F: 5'TCTTCCACCCTGAGCAGCTC3'<br>R: 5' AACCTTGGAGACCAGTGCAG3'

Supplementary Table S3: Soil analysis report showing iron and zinc content (by SGS India Pvt. Ltd.). Mean values ± SEM are shown (n=3 technical replicates)

Analysis	Result
Iron	64721 ± 0.002 mg/gm
Zinc	84.08 ± 0.004 mg/gm
pH (1:5) soil suspension	8.04
Total organic solution	0.75%
Available phosphorus	19.09 ± 0.023mg/kg
Available potassium	3502.71 ± 0.012 mg/kg
Available nitrogen	0.04 %

Supplementary Table *S4*: Concentrations of Iron and Zinc in WT, TF1and TF2 plants

Micronutrients	Tissues	Developmental Stages	WT			TF1			TF2		
Iron	Roots		Mean	SEM	Repl.	Mean	SEM	Repl.	Mean	SEM	Repl.
		Vegetative	44.15	0.502295	3	41.74	0.34641	3	42.36	0.467654	3
		Milk	41.11	0.588897	3	43.49	0.473427	3	43.44	0.232188	3
		Dough	44.46	0.23094	3	34.69	0.404145	3	37.93	0.375278	3
		Matured	38.28	0.271355	3	32.13	0.340637	3	34.73	0.46188	3
	Flag Leaves	Milk	3.68	0.011547	3	3.42	0.017321	3	3.61	0.017321	3
		Dough	3.13	0.017321	3	3.11	0.023094	3	2.85	0.023094	3
		Matured	3.31	0.028868	3	3.26	0.028868	3	3.37	0.017321	3
	Seeds	Milk	6.57	0.173205	3	15.99	0.519615	3	14.02	0.288675	3
		Dough	13.79	0.404145	3	18.21	0.519615	3	16.11	0.23094	3
		Matured	15.7	0.34641	3	20.1	0.69282	3	18.82	0.46188	3
Zinc	Roots	Vegetative	4.4	0.11547	3	4	0.057735	3	3.8	0.028868	3
		Milk	3.6	0.028868	3	4	0.150111	3	3.81	0.029059	3
		Dough	3.8	0.046188	3	3.79	0.043333	3	3.7	0.028868	3
		Matured	4.6	0.051962	3	4.4	0.051962	3	4.5	0.028868	3
	Flag Leaves	Milk	0.99	0.051962	3	1.4	0.034641	3	1.32	0.023094	3
		Dough	2.6	0.057735	3	2.82	0.064291	3	2.52	0.023094	3
		Matured	3.4	0.028868	3	3.76	0.033333	3	3.5	0.057735	3
	Seeds	Milk	13.8	0.57735	3	26.15	1.723398	3	24.38	1.732051	3

		Dough	22.2	1.154701	3	30.6	1.154701	3	28.6	1.154701	3
		Matured	30.1	0.11547	3	33.5	0.288675	3	32.2	0.11547	3

Supplementary Table S5: Known miRNAs differentially expressed in WT and TF roots of rice plants during milk stage of seed development

miR_ID	WT(root) expression	TF1 (root) expression	logFC	logCPM	P value	P-value based Significance
**osa_mir1423**	20	55	-1.160564228	5.976036124	0.018538748	Significance
**osa_mir156b.1**	21	54	-1.257201978	6.002384169	0.01130034	Significance
**osa_mir156c.1**	0	50	7.867053063	4.957876694	0.000339552	Significance
**osa_mir156f.1**	0	18	6.404066093	3.777117603	0.043331447	Significance
**osa_mir156g.1**	0	50	7.867053063	4.957876694	0.000339552	Significance
**osa_mir156h.1**	0	18	6.404066093	3.777117603	0.043331447	Significance
**osa_mir156l**	8	0	-7.846396933	3.343484015	0.000667207	Significance
**osa_mir156l.1**	0	18	6.404066093	3.777117603	0.043331447	Significance
**osa_mir160a**	32	113	-0.801761282	6.851736429	0.036383081	Significance
**osa_mir160b**	32	113	-0.801761282	6.851736429	0.036383081	Significance
**osa_mir160c**	32	113	-0.801761282	6.851736429	0.036383081	Significance
**osa_mir160d**	32	112	-0.814560851	6.845448392	0.036383081	Significance
**osa_mir160e**	13	18	-2.140357524	4.878084215	0.001177692	Significance
**osa_mir162b**	0	21	6.624022796	3.948082301	0.024634949	Significance
**osa_mir164e**	6	0	-7.433447471	2.996963879	0.004642655	Significance
**osa_mir166c**	19910	183268	0.579278379	16.85886644	0.004766754	Significance
**osa_mir166d.1**	11	14	-2.257762759	4.579543833	0.002682185	Significance
**osa_mir166h**	132	407	-0.998239	8.859650641	0.000108532	Significance
**osa_mir166i**	112	367	-0.910433602	8.652161828	0.00062833	Significance
**osa_mir166j**	19886	183195	0.580443712	16.85782483	0.004683625	Significance
**osa_mir169c**	0	18	6.404066093	3.777117603	0.043331447	Significance
**osa_mir169g**	0	25	6.873219399	4.145356569	0.007880255	Significance
**osa_mir169h**	0	32	7.22666868	4.430413011	0.00572059	Significance
**osa_mir169j**	0	32	7.22666868	4.430413011	0.00572059	Significance
**osa_mir169k**	0	32	7.22666868	4.430413011	0.00572059	Significance
**osa_mir169l**	0	32	7.22666868	4.430413011	0.00572059	Significance
**osa_mir169m**	0	32	7.22666868	4.430413011	0.00572059	Significance
**osa_mir171b**	0	21	6.624022796	3.948082301	0.024634949	Significance
**osa_mir171c**	0	19	6.481171714	3.836677669	0.043331447	Significance

**osa_mir171d**	0	19	6.481171714	3.836677669	0.043331447	Significance
**osa_mir171e**	0	19	6.481171714	3.836677669	0.043331447	Significance
**osa_mir171f**	0	20	6.554364616	3.89359254	0.024634949	Significance
**osa_mir1861e**	12	20	-1.874903755	4.871316549	0.004295849	Significance
**osa_mir1861k**	12	20	-1.874903755	4.871316549	0.004295849	Significance
**osa_mir1861m**	12	20	-1.874903755	4.871316549	0.004295849	Significance
**osa_mir1862e**	0	21	6.624022796	3.948082301	0.024634949	Significance
**osa_mir1876**	11	9	-2.883115966	4.35530296	0.000243751	Significance
**osa_mir1878**	0	38	7.4730745	4.631988849	0.002009464	Significance
**osa_mir2863b**	0	20	6.554364616	3.89359254	0.024634949	Significance
**osa_mir2871a**	0	21	6.624022796	3.948082301	0.024634949	Significance
**osa_mir3979**	117	1166	0.693712466	9.539787987	0.00516547	Significance
**osa_mir3979.1**	0	39	7.510340985	4.662642285	0.001187534	Significance
**osa_mir399a**	0	36	7.395523027	4.568332417	0.002009464	Significance
**osa_mir399b**	0	36	7.395523027	4.568332417	0.002009464	Significance
**osa_mir399c**	0	36	7.395523027	4.568332417	0.002009464	Significance
**osa_mir399d**	12	171	1.207401726	6.742999799	0.009749302	Significance
**osa_mir399i**	0	39	7.510340985	4.662642285	0.001187534	Significance
**osa_mir408.1**	0	18	6.404066093	3.777117603	0.043331447	Significance
**osa_mir5072**	29	57	-1.64450361	6.32696923	0.000202378	Significance
**osa_mir5144**	0	36	7.395523027	4.568332417	0.002009464	Significance
**osa_mir531a**	23	62	-1.189668189	6.171645034	0.010578883	Significance
**osa_mir531b**	22	52	-1.378434991	6.016043398	0.005758911	Significance
**osa_mir531c**	23	62	-1.189668189	6.171645034	0.010578883	Significance
**osa_mir5504**	9	0	-8.01562525	3.49992286	0.000352487	Significance
**osa_mir5508**	10	7	-3.099049784	4.14834075	0.000252789	Significance
**osa_mir6248**	7	0	-7.654647098	3.176439287	0.002421289	Significance
**osa_mir820a**	31	112	-0.768807879	6.821251378	0.049434205	Significance
**osa_mir820b**	31	112	-0.768807879	6.821251378	0.049434205	Significance
**osa_mir820c**	31	112	-0.768807879	6.821251378	0.049434205	Significance
**osa_mir1423.1**	0	15	6.144434799	3.579862808	0.131529066	Non-significance
**osa_mir1425**	68	306	-0.45291236	8.124312882	0.125299611	Non-significance
**osa_mir1432**	44	222	-0.287873489	7.578363796	0.384695527	Non-significance
**osa_mir156a**	148	1202	0.398575105	9.695131072	0.100128371	Non-significance

**osa_mir156b**	149	1210	0.398430679	9.704674654	0.102288532	Non-significance
**osa_mir156c**	149	1210	0.398430679	9.704674654	0.102288532	Non-significance
**osa_mir156d**	853	2268	-1.212229801	11.50657851	1.70646567503085e-08	Non-significance
**osa_mir156e**	148	1202	0.398575105	9.695131072	0.100128371	Non-significance
**osa_mir156f**	851	2264	-1.21138977	11.50343622	1.7494555361053e-08	Non-significance
**osa_mir156g**	149	1210	0.398430679	9.704674654	0.102288532	Non-significance
**osa_mir156h**	851	2264	-1.21138977	11.50343622	1.7494555361053e-08	Non-significance
**osa_mir156i**	148	1202	0.398575105	9.695131072	0.100128371	Non-significance
**osa_mir156j**	851	2264	-1.21138977	11.50343622	1.7494555361053e-08	Non-significance
**osa_mir156j.1**	5	34	0.141483482	4.829887576	1	Non-significance
**osa_mir159b**	2963	8060	-1.179367848	13.31765644	1.74544724330235e-08	Non-significance
**osa_mir159c**	59	36	-3.328088094	7.027377173	7.12947417758628e-18	Non-significance
**osa_mir159d**	59	36	-3.328088094	7.027377173	7.12947417758628e-18	Non-significance
**osa_mir159e**	60	36	-3.352321421	7.051554747	3.55090085493078e-18	Non-significance
**osa_mir159f**	834	1316	-1.964894977	11.27991005	6.50595152401615e-19	Non-significance
**osa_mir162a**	14	264	1.611510322	7.248576139	9.26417432041123e-05	Non-significance
**osa_mir164a**	0	14	6.046355149	3.50680074	0.131529066	Non-significance
**osa_mir164b**	0	14	6.046355149	3.50680074	0.131529066	Non-significance
**osa_mir164d**	0	9	5.421001942	3.063621883	0.381172953	Non-significance
**osa_mir164f**	0	14	6.046355149	3.50680074	0.131529066	Non-significance
**osa_mir166a**	34387	208501	-0.022991396	17.31715773	0.910520633	Non-significance
**osa_mir166a.1**	12	61	-0.276448932	5.73446443	0.625860156	Non-significance
**osa_mir166b**	34375	208453	-0.02282002	17.31673922	0.911185272	Non-significance
**osa_mir166b.1**	0	14	6.046355149	3.50680074	0.131529066	Non-significance
**osa_mir166d**	34387	208501	-0.022991396	17.31715773	0.910520633	Non-significance
**osa_mir166e**	41	250	-0.014865556	7.627604643	1	Non-significance
**osa_mir166e.1**	10	59	-0.06217378	5.618809246	1	Non-significance
**osa_mir166f**	34387	208501	-0.022991396	17.31715773	0.910520633	Non-significance
**osa_mir166g**	16061	27946	-1.824022514	15.58858833	5.6298987472496e-18	Non-significance
**osa_mir166l**	22	29	-2.216188112	5.673431096	2.91205658924583e-05	Non-significance
**osa_mir166m**	550	1289	-1.394212682	10.81549683	2.9333086137518e-10	Non-significance
**osa_mir167a**	10	115	0.898119157	6.276633344	0.101374984	Non-significance
**osa_mir167b**	10	116	0.910586883	6.285583951	0.101374984	Non-significance
**osa_mir167c**	10	115	0.898119157	6.276633344	0.101374984	Non-significance

**osa_mir167d**	285	2202	0.326634278	10.59296001	0.147692065	Non-significance
**osa_mir167e**	200	1208	-0.028556976	9.892152901	0.906074233	Non-significance
**osa_mir167f**	285	2202	0.326634278	10.59296001	0.147692065	Non-significance
**osa_mir167g**	285	2199	0.324667606	10.59184673	0.149805194	Non-significance
**osa_mir167h**	285	2200	0.325323461	10.59221792	0.149805194	Non-significance
**osa_mir167i**	200	1207	-0.029751542	9.891539222	0.906074233	Non-significance
**osa_mir167j**	285	2202	0.326634278	10.59296001	0.147692065	Non-significance
**osa_mir168a**	520	4110	0.359422945	11.47608779	0.095849986	Non-significance
**osa_mir168a.1**	44	174	-0.638960692	7.399500854	0.061780405	Non-significance
**osa_mir169a**	0	16	6.236268977	3.64902061	0.075793071	Non-significance
**osa_mir169b**	0	17	6.322605682	3.714661285	0.075793071	Non-significance
**osa_mir169e**	0	17	6.322605682	3.714661285	0.075793071	Non-significance
**osa_mir171a**	0	8	5.25528002	2.953758191	0.253731343	Non-significance
**osa_mir171h**	0	17	6.322605682	3.714661285	0.075793071	Non-significance
**osa_mir1846a**	0	5	4.599695542	2.556360226	0.502487562	Non-significance
**osa_mir1846b**	0	5	4.599695542	2.556360226	0.502487562	Non-significance
**osa_mir1862d**	13	57	-0.489095819	5.71719255	0.387478405	Non-significance
**osa_mir1870**	0	5	4.599695542	2.556360226	0.502487562	Non-significance
**osa_mir1883a**	13	5	-3.944728075	4.345736418	1.35519686099381e-06	Non-significance
**osa_mir2055**	0	14	6.046355149	3.50680074	0.131529066	Non-significance
**osa_mir2871a.1**	0	8	5.25528002	2.953758191	0.253731343	Non-significance
**osa_mir2871b**	0	8	5.25528002	2.953758191	0.253731343	Non-significance
**osa_mir2876**	0	14	6.046355149	3.50680074	0.131529066	Non-significance
**osa_mir319b**	495	317	-3.265179445	10.32036183	8.98322255610018e-41	Non-significance
**osa_mir390**	0	13	5.941118147	3.429380433	0.131529066	Non-significance
**osa_mir393a**	0	5	4.599695542	2.556360226	0.502487562	Non-significance
**osa_mir393b**	0	5	4.599695542	2.556360226	0.502487562	Non-significance
**osa_mir393b.1**	0	5	4.599695542	2.556360226	0.502487562	Non-significance
**osa_mir394**	0	13	5.941118147	3.429380433	0.131529066	Non-significance
**osa_mir396a**	20	140	0.183942925	6.735238396	0.698667552	Non-significance
**osa_mir396b**	20	140	0.183942925	6.735238396	0.698667552	Non-significance
**osa_mir396c**	5	60	0.956975858	5.412084874	0.193029032	Non-significance
**osa_mir396d**	47	213	-0.442604954	7.593288578	0.184724875	Non-significance
**osa_mir396e**	746	2080	-1.143692906	11.33385846	1.21762145717322e-07	Non-significance

**osa_mir396f**	746	2075	-1.147164738	11.33274964	1.11811113604945e-07	Non-significance
**osa_mir396g**	47	215	-0.429135263	7.600140835	0.204015188	Non-significance
**osa_mir396h**	47	215	-0.429135263	7.600140835	0.204015188	Non-significance
**osa_mir397a**	124	116	-2.717059439	8.310417307	1.61877012435235e-20	Non-significance
**osa_mir397b**	11	0	-8.304117907	3.786283885	2.86151638512891e-05	Non-significance
**osa_mir398b**	347	576	-1.891583932	10.0148622	6.75810887906774e-16	Non-significance
**osa_mir399h**	0	9	5.421001942	3.063621883	0.381172953	Non-significance
**osa_mir399j**	0	59	8.104896974	5.15624866	4.67603005110771e-05	Non-significance
**osa_mir399k**	0	8	5.25528002	2.953758191	0.253731343	Non-significance
**osa_mir408**	344	1010	-1.069074948	10.23436895	2.54721176769709e-06	Non-significance
**osa_mir5082**	12	6	-3.576489587	4.298250978	2.5995139788154e-05	Non-significance
**osa_mir5083**	0	13	5.941118147	3.429380433	0.131529066	Non-significance
**osa_mir528**	48	303	0.035075718	7.880507756	0.965557841	Non-significance
**osa_mir528.1**	0	9	5.421001942	3.063621883	0.381172953	Non-significance
**osa_mir530**	5	9	-1.751079379	3.722789971	0.102725028	Non-significance
**osa_mir535**	7	21	-1.030624302	4.53693648	0.235318227	Non-significance
**osa_mir535.1**	0	79	8.524707401	5.508667602	3.8531215035095e-06	Non-significance
**osa_mir6250**	30	17	-3.426077501	5.860255522	6.47614853651625e-11	Non-significance
**osa_mir812n**	0	5	4.599695542	2.556360226	0.502487562	Non-significance
**osa_mir812t**	0	5	4.599695542	2.556360226	0.502487562	Non-significance
**osa_mir812u**	0	5	4.599695542	2.556360226	0.502487562	Non-significance
**osa_mir827**	94	176	-1.717041499	8.114517962	3.09074100976033e-09	Non-significance

Supplementary Table S6: Novel miRNAs differentially expressed in WT and TF roots of rice plants during milk stage

miR_ID	WT raw count	TF raw count	logFC	logCPM	PValue	P-value based Significance
**novel_MiR_11**	333	138	0.514131175	15.48330797	0.045591244	Significance
**novel_MiR_15**	32	0	-7.376224655	11.45646716	0.00041343	Significance
**novel_MiR_2**	14	0	-6.194701479	10.53571348	0.043331447	Significance
**novel_MiR_21**	23	0	-6.903180623	11.07710174	0.002503814	Significance
**novel_MiR_22**	0	9	7.331660499	10.39666319	0.000667207	Significance
**novel_MiR_24**	32	0	-7.376224655	11.45646716	0.00041343	Significance
**novel_MiR_26**	333	138	0.514131175	15.48330797	0.045591244	Significance
**novel_MiR_28**	23	0	-6.903180623	11.07710174	0.002503814	Significance
**novel_MiR_30**	333	138	0.514131175	15.48330797	0.045591244	Significance
**novel_MiR_31**	333	138	0.514131175	15.48330797	0.045591244	Significance
**novel_MiR_33**	32	0	-7.376224655	11.45646716	0.00041343	Significance
**novel_MiR_34**	26	0	-7.078666936	11.21646762	0.000794618	Significance
**novel_MiR_38**	23	0	-6.903180623	11.07710174	0.002503814	Significance
**novel_MiR_9**	23	0	-6.903180623	11.07710174	0.002503814	Significance
**novel_MiR_1**	0	15	8.06503915	11.07338118	2.47519426677382e-06	Non-significance

**novel_MiR_10**	0	16	8.15781182	11.16659505	1.35519686099381e-06	Non-significance
**novel_MiR_12**	0	21	8.548925981	11.57525076	2.21220691621053e-08	Non-significance
**novel_MiR_13**	11	0	-5.852139904	10.28954277	0.131529066	Non-significance
**novel_MiR_14**	333	0	-10.74773227	14.37510459	1.38028702588682e-26	Non-significance
**novel_MiR_16**	0	38	9.402811758	12.5264964	5.41923861337886e-14	Non-significance
**novel_MiR_17**	470	0	-11.24462626	14.83331278	2.75764929881476e-33	Non-significance
**novel_MiR_18**	470	0	-11.24462626	14.83331278	2.75764929881476e-33	Non-significance
**novel_MiR_19**	73	0	-8.56116375	12.44562524	1.83960190797737e-08	Non-significance
**novel_MiR_20**	39	0	-7.660067401	11.68907731	4.99870827618896e-05	Non-significance
**novel_MiR_23**	0	219	11.92790874	15.30877094	1.9158864801219e-54	Non-significance
**novel_MiR_25**	415	157	0.382733003	15.7282977	0.131258573	Non-significance
**novel_MiR_27**	0	11	7.619537793	10.6488794	9.96168072903945e-05	Non-significance
**novel_MiR_29**	470	0	-11.24462626	14.83331278	2.75764929881476e-33	Non-significance
**novel_MiR_3**	2962	0	-13.89996492	17.38466745	1.74649987321561e-88	Non-significance
**novel_MiR_32**	0	12	7.744457204	10.76386049	5.32834085506765e-05	Non-significance
**novel_MiR_35**	10	0	-5.717131718	10.19594464	0.131529066	Non-significance
**novel_MiR_36**	0	16	8.15781182	11.16659505	1.35519686099381e-06	Non-significance
**novel_MiR_37**	470	0	-11.24462626	14.83331278	2.75764929881476e-33	Non-significance

**novel_MiR_39**	0	27	8.910639796	11.97129561	2.45933802464118e-10	Non-significance
**novel_MiR_4**	0	16	8.15781182	11.16659505	1.35519686099381e-06	Non-significance
**novel_MiR_40**	407	155	0.39231263	15.70544294	0.11455195	Non-significance
**novel_MiR_41**	333	0	-10.74773227	14.37510459	1.38028702588682e-26	Non-significance
**novel_MiR_5**	0	895	13.95858586	17.39797622	3.2917517262233e-108	Non-significance
**novel_MiR_6**	310	368	2.031993145	16.43048772	1.8945867931324e-17	Non-significance
**novel_MiR_7**	0	27	8.910639796	11.97129561	2.45933802464118e-10	Non-significance
**novel_MiR_8**	470	0	-11.24462626	14.83331278	2.75764929881476e-33	Non-significance

Supplementary Table S7: (A) novel miRNAs identified in WT roots and (B) TF roots, and corresponding loci position on different chromosome

(A)

miR ID	score	chromosome	strand	hairPin_loci	Expression (number of mature reads)	mature_loci
miR3	1510.12	chr1	0	35776812-35776916	2962	35776889-35776906
miR2	6.03	chr1	0	35033156-35033260	14	35033233-35033250
miR6	185.51	chr2	0	10335273-10335381	310	10335349-10335371
miR9	11.91	chr4	0	597935-598082	23	598054-598072
miR8	3.63	chr4	0	597932-598089	470	598059-598079
miR11	3.54	chr4	0	26753212-26753410	333	26753380-26753400
miR15	16.69	chr6	0	17137406-17137539	32	17137416-17137436
miR18	4.25	chr6	0	23376285-23376449	470	23376419-23376439
miR17	4.18	chr6	0	23376283-23376447	470	23376293-23376313
miR13	3.77	chr6	0	7558731-7558946	11	7558741-7558762
miR14	3.65	chr6	0	14026788-14026980	333	14026798-14026818
miR19	46.9	chr7	0	137463-137653	73	137623-137643
miR20	22.74	chr7	0	23277667-23277820	39	23277792-23277810
miR21	11.85	chr7	0	23277669-23277822	23	23277679-23277697
miR24	16.73	chr8	0	14748232-14748365	32	14748335-14748355
miR26	3.64	chr8	0	23874703-23874901	333	23874871-23874891
miR25	3.5	chr8	0	17556697-17556806	415	17556776-17556796
miR30	174.18	chr10	0	21279127-21279318	333	21279137-21279157
miR31	174.14	chr10	0	21279129-21279320	333	21279290-21279310
miR28	12.07	chr10	0	3019371-3019472	23	3019381-3019399
miR29	3.68	chr10	0	17480901-17481065	470	17481035-17481055
miR33	16.72	chr11	0	5001092-5001225	32	5001102-5001122
miR38	15.44	chr11	0	21857766-21857920	23	21857892-21857910
miR37	4.32	chr11	0	21857763-21857927	470	21857897-21857917
miR34	3.93	chr11	0	19306972-19307192	26	19306982-19307002
miR35	3.6	chr11	0	19306976-19307186	10	19306986-19307002
miR41	3.8	chr12	0	10057064-10057268	333	10057074-10057094
miR40	3.56	chr12	0	7127003-7127134	407	7127013-7127033

(B)

miR_ID	score	chromosome	strand	hairPin_loci	Expression (number of mature reads)	mature_loci
miR1	6.08	chr1	0	11747521-11747709	15	11747531-11747553
miR5	464.7	chr2	0	5569887-5569988	895	5569897-5569915
miR6	285.44	chr2	0	10335273-10335381	368	10335349-10335371
miR4	6.49	chr2	0	4360326-4360515	16	4360336-4360358
miR7	13.07	chr3	0	32216706-32216896	27	32216716-32216738
miR10	6.6	chr4	0	7146811-7146999	16	7146967-7146989
miR11	3.54	chr4	0	26753212-26753410	138	26753380-26753400
miR16	25.47	chr6	0	20890870-20890980	38	20890950-20890970
miR12	10.45	chr6	0	203650-203743	21	203713-203733
miR23	100.81	chr8	0	11763807-11763902	219	11763870-11763892
miR27	7.96	chr8	0	27121091-27121172	11	27121101-27121121
miR22	4.82	chr8	0	3736639-3736722	9	3736649-3736670
miR26	3.64	chr8	0	23874703-23874901	138	23874871-23874891
miR25	3.5	chr8	0	17556697-17556806	157	17556776-17556796
miR30	3.67	chr10	0	21279127-21279318	138	21279137-21279157
miR31	3.63	chr10	0	21279129-21279320	138	21279290-21279310
miR39	12.65	chr11	0	23266761-23266950	27	23266919-23266940
miR32	8.7	chr11	0	2880367-2880555	12	2880524-2880545
miR36	6.68	chr11	0	20815483-20815672	16	20815493-20815515
miR40	3.56	chr12	0	7127003-7127134	155	7127013-7127033

Supplementary Table S8: qRT-PCR mediated validation of expression of novel miRNAs in TF1 and TF2 plants

		WT			TF1			TF2		
		Mean	SEM	Rep.	Mean	SEM	Rep	Mean	SEM	Rep.
	miR2	6.27	0.98	3	5.73	1.29	3	5.08	1.07	
Roots of vegetative stage	miR9, miR21, miR28, miR38	4.11	1.02	3	5.69	1.11	3	6.11	1.66	3
	miR11, miR26, miR30, miR31	1.98	0.52	3	1.51	0.88	3	1.49	0.84	3
	miR15, miR24 miR33	7.75	0.47	3	0.03	0.0005	3	0.05	0.0007	3
	miR22	0.58	0.028	3	1.55	0.59	3	1.36	0.98	3
	miR34	10.52	1.96	3	4.43	1.23	3	4.22	1.51	3
Roots of milk stage	miR2	14.1	1.98	3	0.15	0.02	3	0.22	0.073	3
	miR9, miR21, miR28, miR38	12	1.67	3	0.6	0.054	3	1.32	0.61	3
	miR11, miR26, miR30, miR31	17	1.18	3	3.5	0.74	3	5.93	1.45	3
	miR15, miR24 miR33	5	0.92	3	0.00005	0.000007	3	0.00009	0.000008	3
	miR22	0.8	0.0288	3	3	0.127	3	2.3	0.1732	3
	miR34	7	0.82	3	1.8	0.89	3	2.62	1.3	3
Flag leaves of milk stage	miR2	0.78	0.08	3	0.24	0.03	3	0.38	0.05	3
	miR9, miR21, miR28, miR38	2.5	0.7	3	0.14	0.04	3	0.22	0.06	3
	miR11, miR26, miR30, miR31	2	0.6	3	0.36	0.02	3	0.46	0.04	3
	miR15, miR24 miR33	0.07	0.005	3	6	0.95	3	5.1	0.81	3
	miR22	4	0.9	3	0.9	0.14	3	1.22	0.3	3
	miR34	2	0.69	3	0.7	0.1	3	0.92	0.25	3
Seeds of	miR2	0.7	0.094	3	1.2	0.38	3	1.6	0.29	3
	miR9,	0.1	0.032	3	0.03	0.0079	3	0.04	0.004	3

milk stage	miR21, miR28, miR38									
	miR11, miR26, miR30, miR31	0.1	0.019	3	0.22	0.071	3	0.19	0.0569	3
	miR15, miR24 miR33	0.01	0.005	3	0.024	0.009	3	0.028	0.0076	3
	miR22	0.005	0.0003	3	0.02	0.004	3	0.018	0.0017	3
	miR34	0.32	0.051	3	0.4	0.037	3	0.33	0.042	3

Supplementary Table S9: qRT-PCR mediated validation of expression of target genes in TF1 and TF2 plants

Tissues	Target genes	WT			TF1			TF2		
		Mean	SEM	Repl.	Mean	SEM	Repl.	Mean	SEM	Repl.
Roots of veg. stage	TG of miR2	0.45	0.023094	3	0.53	0.012702	3	0.49	0.011547	3
	TG of miR9, miR21, miR28, miR38	0.37	0.017321	3	0.004	0.000115	3	0.013	0.001155	3
	TG of miR11, miR26, miR30, miR31	0.112	0.014048	3	0.46	0.044456	3	0.42	0.023094	3
	TG of miR15, miR24, miR33	0.003	0.000115	3	0.02	0.002309	3	0.025	0.001732	3
	TG of miR22	0.004	0.000346	3	0.0004	0.0000005	3	0.00032	0.0000001	3
	TG of miR34	0.002	0.000404	3	0.4	0.290057	3	0.25	0.011547	3
Roots of milk stage	TG of miR2	0.006	0.000173	3	0.048	0.004619	3	0.052	0.002309	3
	TG of miR9, miR21, miR28, miR38	1	0.057735	3	17.42	1.154701	3	17.86	1.154701	3
	TG of miR11, miR26, miR30, miR31	0.107	0.091537	3	65.24	1.735291	3	55.39	1.340178	3

	TG of miR15, miR24, miR33	0.67	0.040415	3	1	0.057735	3	0.78	0.011547	3
	TG of miR22	1	0.057735	3	0.41	0.028868	3	0.29	0.017321	3
	TG of miR34	0.0115	0.001756	3	0.32	0.011547	3	0.47	0.040415	3
Flag leaves of milk stage	TG of miR2	0.03	0.002887	3	0.51	0.057735	3	0.41	0.028868	3
	TG of miR9, miR21, miR28, miR38	0.26	0.034641	3	2.99	0.057735	3	3.08	0.288675	3
	TG of miR11, miR26, miR30, miR31	0.11	0.017321	3	0.6	0.017321	3	0.28	0.034641	3
	TG of miR15, miR24, miR33	1.7	0.11547	3	0.62	0.028868	3	0.055	0.002887	3
	TG of miR22	0.002	0.000005	3	0.0115	0.001756	3	0.035	0.002887	3
	TG of miR34	0.77	0.028868	3	1.43	0.017321	3	1.35	0.057735	3
Seeds of milk stage	TG of miR2	3.76	0.11547	3	0.00109	0.000105	3	0.008733	0.000636	3
	TG of miR9, miR21, miR28, miR38	1	0.11547	3	8.87	0.57735	3	8.51	0.57735	3

	TG of miR11, miR26, miR30, miR31	9.5	1.127845	3	1	0.063509	3	0.92	0.040415	3
	TG of miR15, miR24, miR33	1.17	0.11547	3	0.26	0.12252	3	0.05	0.002887	3
	TG of miR22	0.055	0.002887	3	0.033	0.001732	3	0.040667	0.002603	3
	TG of miR34	1.25	0.11547	3	0.034	0.002309	3	0.025	0.002887	3

Supplementary Table S10: qRT-PCR mediated validation of expression of transporters in TF1 and TF2 plants

Tissue	Transporters	WT			TF1			TF2		
		Mean	SEM	Repl.	Mean	SEM	Repl.	Mean	SEM	Repl.
Roots of vegetative stage	OsNRAMP4	0.112	0.014048	3	0.46	0.044456	3	0.42	0.023094	3
	OsYSL2	0	0	3	0	0	3	0	0	3
	OsYSL15	0.048	0.003464	3	1.64	0.083732	3	1.91	0.069282	3
	OsYSL18	1	0.046188	3	1.35	0.051962	3	1.12	0.057735	3
	OsFRO2	1	0.063509	3	0.85	0.040552	3	0.62	0.063509	3
	OsIRT1	1	0.083732	3	1.13	0.040415	3	1.22	0.034641	3
	OsIRT2	0.97	0.028868	3	1.56	0.063509	3	1.61	0.080829	3
Roots of milk stage	OsNRAMP4	0.107	0.091537	3	65.24	1.735291	3	55.39	1.340178	3
	OsYSL2	0.003	0.001862	3	0.004	0.000361	3	0.006	0.000924	3
	OsYSL15	0.86	0.057735	3	45.31	1.766893	3	41.50	1.941686	3
	OsYSL18	1.86	0.040961	3	2.69	0.080829	3	2.13	0.092376	3
	OsFRO2	51.53	1.850225	3	239.69	2.855254	3	210.53	7.067572	3
	OsIRT1	0.94	0.046188	3	2.67	0.063509	3	2.02	0.057735	3
	OsIRT2	50.68	2.163719	3	256.68	2.896068	3	207.63	4.587085	3
Flag leaves of milk stage	OsNRAMP4	0.11	0.017321	3	0.6	0.017321	3	0.28	0.034641	3
	OsYSL2	.185	0.041603	3	0.06	0.006928	3	0.024	0.005774	3
	OsYSL15	0.32	0.051962	3	0.002	0.000577	3	0.005	0.001155	3
	OsYSL18	0.003	0.001862	3	0.15	0.046188	3	0.25	0.040415	3
	OsFRO2	1.35	0.046188	3	1.79	0.09815	3	1.55	0.040415	3
	OsIRT1	0.043	0.011667	3	0.103	0.007126	3	0.094	0.006351	3
	OsIRT2	1.58	0.051962	3	0.8	0.063509	3	0.65	0.051962	3
Seeds of milk stage	OsNRAMP4	9.5	1.127845	3	1	0.063509	3	0.92	0.040415	3
	OsYSL2	1.07	0.034641	3	1	0.040415	3	1.12	0.057735	3
	OsYSL15	12.67	1.042151	3	0.04	0.006351	3	0.032	0.005196	3
	OsYSL18	1.23	0.109697	3	1	0.023094	3	0.97	0.046188	3
	OsFRO2	30.61	1.197762	3	0.13	0.028868	3	0.09	0.023094	3
	OsIRT1	11.15	0.497427	3	1	0.121244	3	1.08	0.063509	3
	OsIRT2	30.55	1.798342	3	0.004	0.001732	3	0.006	0.002887	3

Supplementary Figure S1: Venn diagram of known miRNAs exhibiting 26 differentially expressed miRNAs among 59 significant miRNAs

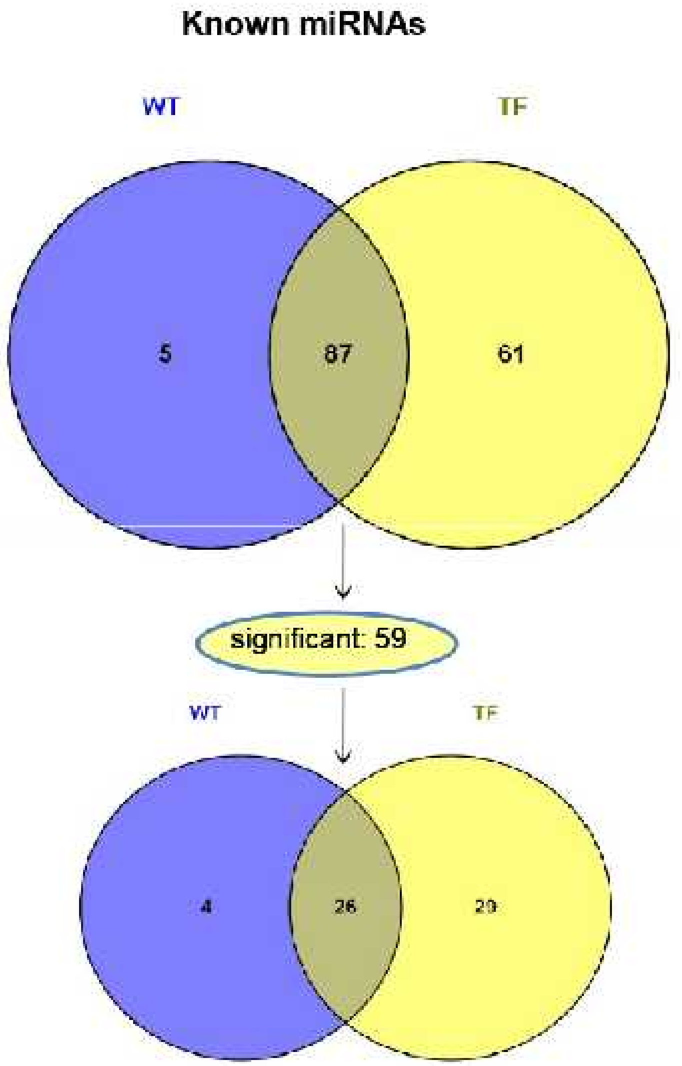

Supplementary Figure S2: Nucleotide sequences of 14 hairpin forming miRNA precursors representing significantly expressed novel matured miRNAs in WT and TF roots of rice plants. The brackets indicate base pairing and dots represent mismatches in the precursor sequences. The red colour in nucleotide sequences denotes the matured miRNA sequence.

Novel_miR2:
Ggcgccggtcgccgcgtcgtcgtcgtcgtccgggcccgtcgacgctgccggcgaggagctggacgtcatggactacgacatgacggacgcgctgttctgggggcc
.........((.(((((((((((.(((((..((..((((.((((...(((((.....))))).)))))))).))))))))))))).)))))).))...........

Novel_miR9:
ttttccgtccgccttgatcgctattgacccaaccacggatgcgatatgacgtttctacccctcacaagtacaagtagaatgcatggcaatgaggggtagaaacgatgtgcgtggttggatcaatagcga
tcaaggcggatggaaaacg
........((((((((((((((((((.(((((((((.((.........((((((((((((((...((.((.((.....)).))))..))))))))))))))..)).))))))))).))))))))))))))))))..........

Novel_miR15:
ggggcctcccacctgcgggtcttcggctgcctcgcgttcgtcaaggagcttggccacatcgacaagcttgacgacaggagcaccccaggggtgttcatcggctacgcggagggctcgaaggcct
accgcatcct
...........(((.(((((((((.((.(((..)))...)).))))))))).)))............

Novel_miR21:
ttttctgtccgccttgatcgctattgacccagtcacgcacacgatatgatgtttctaccctcattgacatgcattctacttgtacttgtgaggggtagatacgttatatcacgtgtgtggctaggtcaatagcg
atcaaggcgaacgaaaaacg
.........((((((((((((((((((((.((((((((((..((((((((((.(((((((((((.....((((.......))))...)))).))))))).))))))))))..)))))))))).))))))))))))))))))))...........

Novel_miR22:
atttgaaatgaggattagagggaactgaaccattttgatgaatatcagaaggttcagtttcctctaatatctcgccttgaatgg
(((..(..(((((((((((((((((((((((.(((((((....))))))))))))))))))))))))))))))).)))))..)..)))..

Novel_miR24:
ggggtctcccacctgcgggtcttcggctgcctcgtgttcgtcaaggagcttggccccatcggcaagctcgacgacaggagcaccccaggggtgttcatcggctacgcggagggctcgaaggcct
atcacatcct
...........(((.(((((((((.((.(((..)))...)).))))))))).)))...........

Novel_miR28:
ttttccgtccgccttgatcgctattgacccagtcacgcacgcgatatgacgtttctacccctcataggctgggtcaatagcgatcaaggcagacggaaaacg
........(.((((((((((((((((((((((((..............................))))))))))))))))))))))))).)..........

Novel_miR33:
ggggtctcccacctgcgggtcttcggctgcctcgcgttcgtcaaggagcttggccccatcagcaagctcgatgacaggagcaccccaggggtgttcatcggctacgcggagggctcgaaggcct
atcgcatcct
...........(((.(((((((((.((.(((..)))...)).))))))))).)))...........

Novel_miR34:
ttctcacttttaacacttccgtcaattcttgaccgttttccgtctgtcttgattgctattgacccaaccacgcacgcgatatgatgtttctaccctcacaagtacaagtagaatgcatgataatgagggtagaa
atatcatatcgcttgcgtggctagattaatagcgatcaagacgaacgaaaaacgatccagaattgacggaagtgttaaaaatgggaa
.(((((.((((((((((((((((((((((.((.((((((.(((.((((((((((((((((((.(...(((((((.((((((((((((((((((((((((...((.((.((.....)).))))..)))))))))))))))))))))))).)))))))...).))))))))))
)))))))).))).)))))).)).)))))))))))))))))))))).))))).

Novel_miR38:
ttttccgtccgccttgatcgctattgacccagccacgcacgcgatatgacgtttctacccctcattgccatgcattctacttgtacttgtgaggggtagaaccgtcatatcgcgtgcgtagatgggtcaata
gcgatcaaggcggacagaaaacg
........((((((((((((((((((((((.(.(((((((((((((((((.(((((((((((((.....((((.......))))...))))))))))))).))))))))))))))))).).))))))))))))))))))))))..........

Novel_miR11:
gcacgccacatggactatgacatggatttttattttatttttctcccttcttccttctcttttctcttttatgaaaagacgatgacatggacgatgacgtgtataaggcattttcatcttttcgacaaggaagaaa
gagaaaaaaaagaaggaagaagggagaaaaaaatccatgtcatcgtccacgtggcgtgcca
(((((((((.(((((.((((((((((((((((...........((((((((((((((((((.(((((((((((..)))))))))))...))))))))))))))))))))))))))))))))).
))))).)))))))))..

Novel_miR26:
gcacgccatgtggacgatgacatggattttttttctcccttcttctttctcttttctctttaatgaaaatacgatggcatggacgataacgtgtatgaggcattttcatcttttcgacaaggaagaaagagaaa
aaaagaaggaaaaagggagaaaaaataaaataaaaatccatgtcatcgtccacgtggcgtgcca
(((.((((((((.((((((((((..))))))))))..)))))))).)))))))))))........)))))))))))))))
)))))))))))))))..

Novel_miR30:
gcacgccacgtggacgatgacatggatttttattttattttttctcctttcttccttcttttttttctctttcttccttgtcgaaaagatgaaaatgcctcatacacgtcatcgtcctttcataaaagagaaaagagaa
ggaagaagggagaaaaataaaaaaaatccatgtcatcgtccacgtggcatgcca
(((.((((((((((((((((((((((((((((..(((((((((.(((((((((((((((((((((.((((((......(((.((((.(((((..(((.........)))..))))).)))
))))))))))))))))).)))..

Novel_miR31
gcatgccacgtggacgatgacatggatttttttttatttttctcccttcttccttctcttttctcttttatgaaaggacgatgacgtgtatgaggcattttcatcttttcgacaaggaagaaagagaaaaaaagaag
gaagaaaggagaaaaaataaaataaaaatccatgtcatcgtccacgtggcgtgctg
((.(((((((((((.(((((((((((.((((((((.((....((((.....))))..)).))))))))........))))))))))..))))))))))).)))).)))))))))..))))))))))
))))))))))))))))))))..

Supplementary Figure S3: qRT-PCR-mediated validation of 10 known MIR gene expression in (A) root of TF1 plants collected at vegetative stage (B) root of TF1 plants collected at milk stage Known (C) root of TF2 plants collected at vegetative stage (D) root of TF2 plants collected at milk stage (WT, wild type; TF, transgenic ferritin)

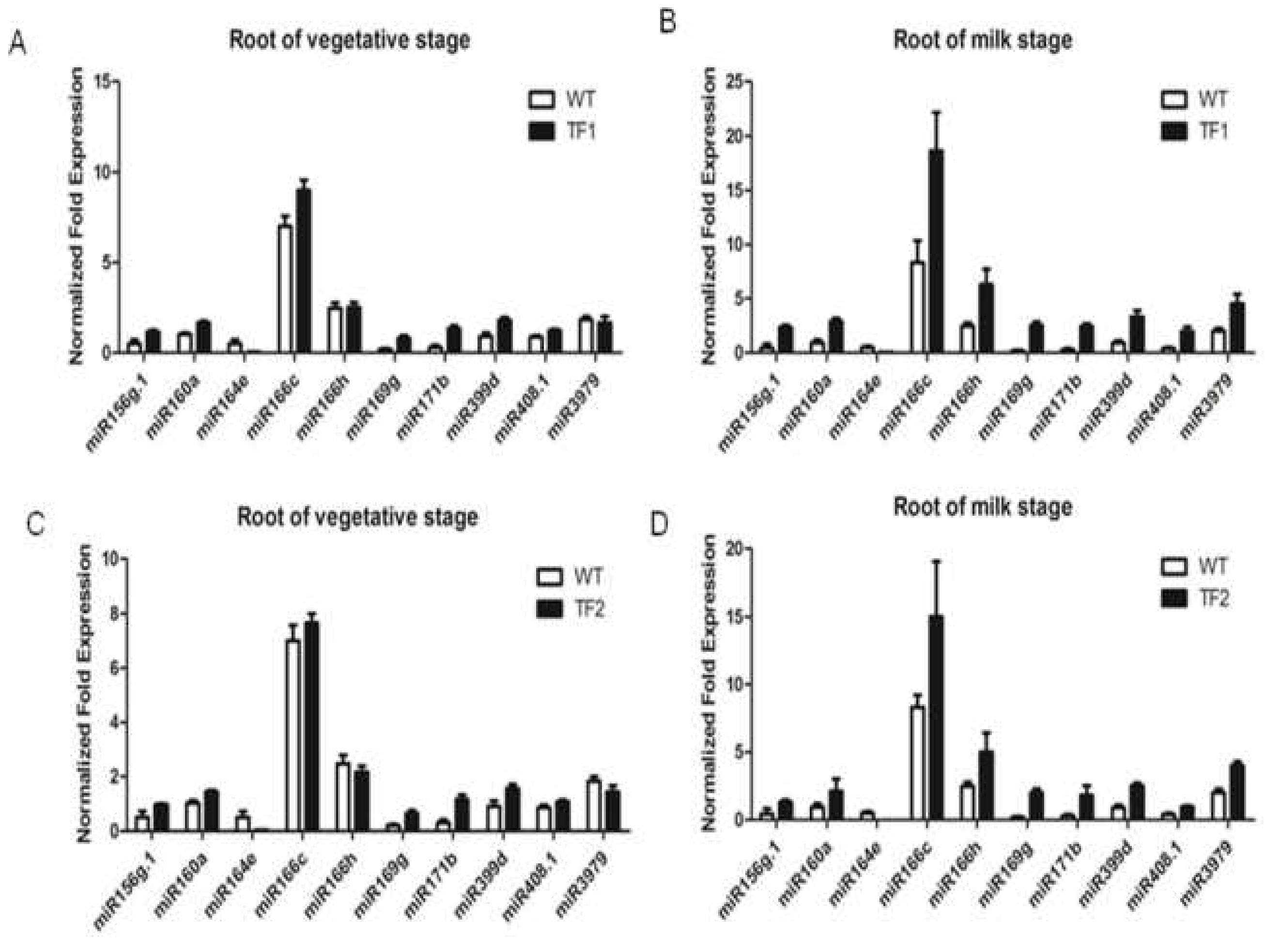

Supplementary Figure S4: qRT-PCR analysis of soy*FER*1 gene expression in shoot of WT, TF1 and TF2 plants during vegetative stage. TF1 showed highest relative transcript level of soy*FER*1 expression. Mean values ± SEM are shown (n=3 biological replicates). Asterisks indicate the significant differences in relative transcript level of soy*FER*1 between WT and two TF plants (**** if P<0.0001, ** if P<0.005; WT, wild type; TF, transgenic ferritin)

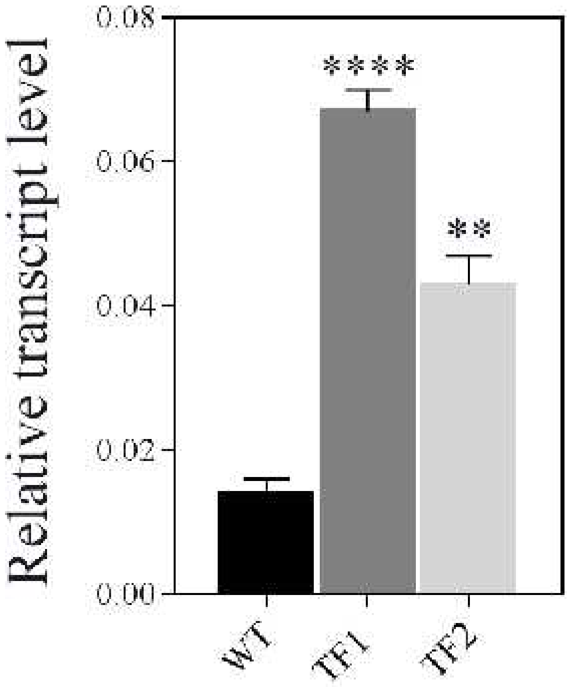

Supplement: Supplementary Data [file supp_erw346_supplementary_tables_S1_S10_figures_S1_S4.pdf]
